# Genome-wide identification and expression analysis of the IQD gene family in moso bamboo (*Phyllostachys edulis*)

**DOI:** 10.1038/srep24520

**Published:** 2016-04-20

**Authors:** Min Wu, Yuan Li, Danmei Chen, Huanlong Liu, Dongyue Zhu, Yan Xiang

**Affiliations:** 1 Key Laboratory of Crop Biology of Anhui Province, School of Life Sciences, Anhui Agricultural University, Hefei 230036, China; 2 Laboratory of Modern Biotechnology, School of Forestry and Landscape Architecture, Anhui Agricultural University, Hefei 230036, China

## Abstract

Members of the plant-specific IQ67-domain (IQD) protein family are involved in various aspects of normal plant growth and developmental processes as well as basal defence response. Although hundreds of IQD proteins have been identified, only a small number of IQDs have been functionally characterized. Moreover, no systematic study has been performed on moso bamboo. In this study, we performed for the first time a genome-wide identification and expression analysis of the IQD gene family in moso bamboo. We identified 29 non-redundant *PeIQD* encoding genes. Analysis of the evolutionary patterns and divergence revealed that the IQD genes underwent a large-scale event around 12 million years ago and the division times of IQD family genes between moso bamboo and rice, and, between moso bamboo and *Brachypodium*, were found to be 20–35 MYA and 25–40 MYA, respectively. We surveyed the putative promoter regions of the *PeIQD* genes, which showed that largely stress-related *cis*-elements existed in these genes. The expression profiles of the IQD genes shed light on their functional divergence. Additionally, a yeast two-hybrid assay proved that *PeIQD8* can interact with *PeCaM2* and that IQ or I in the IQ motif is required for *PeIQD8* to combine with CaM2.

Ca^2+^ is a pivotal cytosolic second messenger, which plays a prominent role in many essential biological processes in plants. It participates in plant growth and development, photosynthetic electron transport and photophosphorylation, and regulates stomatal aperture and hormonal functions. In addition, it can also prevent plant-pathogen interactions^1^,^2^. When plants are subjected to numerous environmental cues of biotic and abiotic nature and endogenous physiological and developmental conditions, they generate intracellular calcium transients, and decoding of calcium signatures, and the transformation of the signal into cellular responses are integral parts of the transduction process^3^,^4^. Calcium spikes are recognized by several Ca^2+^-binding proteins and are decoded via Ca^2+^-dependent conformational changes in these sensor polypeptides and interacting target proteins^1^,^2^,^3^,^4^. Several classes of Ca^2+^ sensors have been identified in plants that contain a Ca^2+^-binding helix-loop-helix fold known as the “EF-hand motif”. Based on the number of EF-hand motifs, Ca^2+^ sensors are classified into four classes: calmodulin (CaM), containing four EF-hand motifs; calcineurin B-like (CBL) proteins, possessing three EF-hand motifs; Ca^2+^-dependent protein kinases (CDPKs), containing four EF-hand motifs and a Ca^2+^-dependent Ser/Thr protein kinase domain, and the last type – Ca^2+^ sensors lacking EF-hand motifs^4^,^5^.

Among these Ca^2+^-binding proteins, calmodulin (CaM) has been the most extensively studied. Calmodulin is small, acid resistant, heat resistant and highly stable^3^,^4^,^5^. This multifunctional protein has been found in higher plants and shares various functions with protein kinases, metabolic enzymes, cytoskeleton-associated proteins, and others^5^. CBL sensor proteins act as the structural bases for Ca^2+^-binding and also have interact specifically with the Ser/Thr protein kinases group, which are designated as CBL-interacting protein kinases (CIPKs)^4^,^5^. CIPKs most likely substitute for CBLs, which have no enzymatic activity, to receive and transmit the Ca^2+^ signals, while calmodulins (CaMs) and CBL sensor proteins have no catalytic activity on their own and are therefore sometimes referred to as “Ca^2+^ sensor relays”, in contrast to CDPK proteins, which are considered “Ca^2+^ sensor responders”. Because of their dual functions (a calmodulin-like domain with four EF-hand motifs and a Ca^2+^-dependency)^5^, CDPK proteins act as the catalytic effectors and play essential roles in hormone and stress signalling pathways as well as in plant responses to pathogens^5^. CaM recruitment sequence motifs contain three specific motifs: an IQ motif, which mediates Ca^2+^-independent CaM retention, and the 1–5–10 and 1–8–14 motifs, which are thought to mediate CaM retention in a Ca^2+^-dependent manner, and which are distinguished by the spacing of their hydrophobic and basic amino acid residues^4^,^5^,^6^. The encoded IQD proteins contain a plant-specific domain of 67 conserved amino acid residues, referred to as the “IQ67 domain”. The IQ67 domain is characterized by the accurate spacing of three copies of the IQ motif (IQxxxRGxxxR) or ([ILV]QxxxRxxxx[R, K])^6^ –which are separated by two short sequences with 11 and 15 amino acid residues, respectively. In addition, each IQ motif partially overlaps with three copies of the 1–8–14 motif ([FILVW] × 6[FAILVW] × 5[FILVW]) and four copies of the 1–5–10 motif ([FILVW] × 3[FILV] × 4[FILVW])^6^,^7^. Another common hallmark of the IQ67 domain is that has a highly conserved exon-intron boundary that interrupts codons 16 and 17 via a phase-0 intron^5^. These features allow the IQ67 domain to fold into a basic amphiphilic helix structure, which enables these proteins to perform specific roles. The first representative CaM target proteins have been identified in *Arabidopsis thaliana* and rice, and encoded 33 and 29 IQD1-like genes, respectively, were identified within the IQ67 domain^6^. Subsequently, they have been identified in other species (soybean, *Brachypodium distachyon*, *Populus trichocarpa*, tomato and others). IQD genes have also been identified in Physcomitrella, though algae contain no any IQD genes, which suggests that IQD proteins originate from an ancient family of CaM/CML-binding proteins that contributed to the early evolution of land plants. Furthermore, the function of IQD genes has been analyzed. For example, *AtIQD1* can stimulate glucosinolate accumulation and regulate plant defence, and *AtIQD22* acts as a negative regulator of the response to the plant hormone gibberellin^5^,^6^,^8^. Genetic analysis has shown that IQD12/SUN from tomato (*Solanum lycopersicum*) controls elongated fruit shape by increasing cell division in a longitudinal direction and decreasing cell division in a transverse direction in the fruit^9^,^10^,^11^. In addition, *GmIQD* III genes are regulated by MeJA (methyljasmonate) stress^12^, and the expression of 12 selected IQD members were regulated by MeJA and PEG treatments (polyethylene glycol electrolyte solution) in *Populus trichocarpa*^13^.

Bamboo is one of the most important non-timber forest products worldwide and comprises over 70 genera and 1200 species^14^. In China, there are about 48 genera and nearly 500 species are distributed in subtropical regions, or south of 40° northern latitude, particularly south of the Yangtze River. Moso bamboo (*Phyllostachys edulis*) is the most important bamboo species in China. It is the most widely distributed with the largest planting area (over two-thirds of the total planted bamboo area) and has great economic importance in China, being used as timber, paper and art ware, and the shoots as delicious food^15^. The availability of a genome sequence provided us with the perfect opportunity to conduct a comprehensive, genome-wide analysis of the IQD genes in moso bamboo.

In the present study, we performed for the first time a comprehensive analysis of the IQD gene family in moso bamboo. We identified 29 non-redundant *PeIQD* encoding genes were identified and systematically analyzed them, studying their phylogenetic relationships, gene structure, conserved motifs, evolutionary patterns and divergence, yeast two-hybrid assay, *cis*-elements and expression profiling. On the basis of the expression profiles of IQD genes and the *cis*-elements in the putative promoter regions analysis in moso bamboo, the functions of *PeIQDs* were predicted. Besides, analysis with a yeast two-hybrid assay revealed that *PeIQD8* can interact with *PeCaM2* and that IQ or I in the IQ motif is required for *PeIQD8* to combine with CaM2. The results of this study provide a biological reference for further elucidating the role of IQDs in plants.

## Results

### Identification of IQD gene family in moso bamboo

In order to conduct a genome-wide identification of the IQD gene families in moso bamboo, the HHM profile of IQ domain (PF00612) was exploited as query to identify the IQD genes in the moso bamboo genome (http://www.ncgr.ac.cn/bamboo, accessed February 2016). We identified 35 putative IQD genes, then these genes were conducted to confirm the existence of the conserved IQD domain with InterproScan. After removing redundant sequences, 29 IQD genes were identified, and all of these predicted that IQD proteins had a typical “IQ calmodulin-binding motif” domain (PF00612) which is a major calcium (Ca^2+^) regulator. We named these 29 IQD genes – IQD1 to IQD29, according to their physical locations (from top to bottom) on chromosomes (Table 1). The identified IQD genes in moso bamboo encode proteins ranging from 190 to 940 amino acids (aa) in length with an average of 486 aa. The other characteristics of the IQD genes –including isoelectric point (pI), molecular weight (MW) chromosome location, and exons – are listed in Table 1. The open reading frame (ORF) length ranged from 573 bp (IQD6) to 2823 bp (IQD17), the MW from 20417.05 (IQD6) to 105765.41 (IQD17) Da, and the pI from 5.02 (IQD5) to 11.12 (IQD9).

### Phylogenetic analysis of the IQD gene family

**Figure 1:**
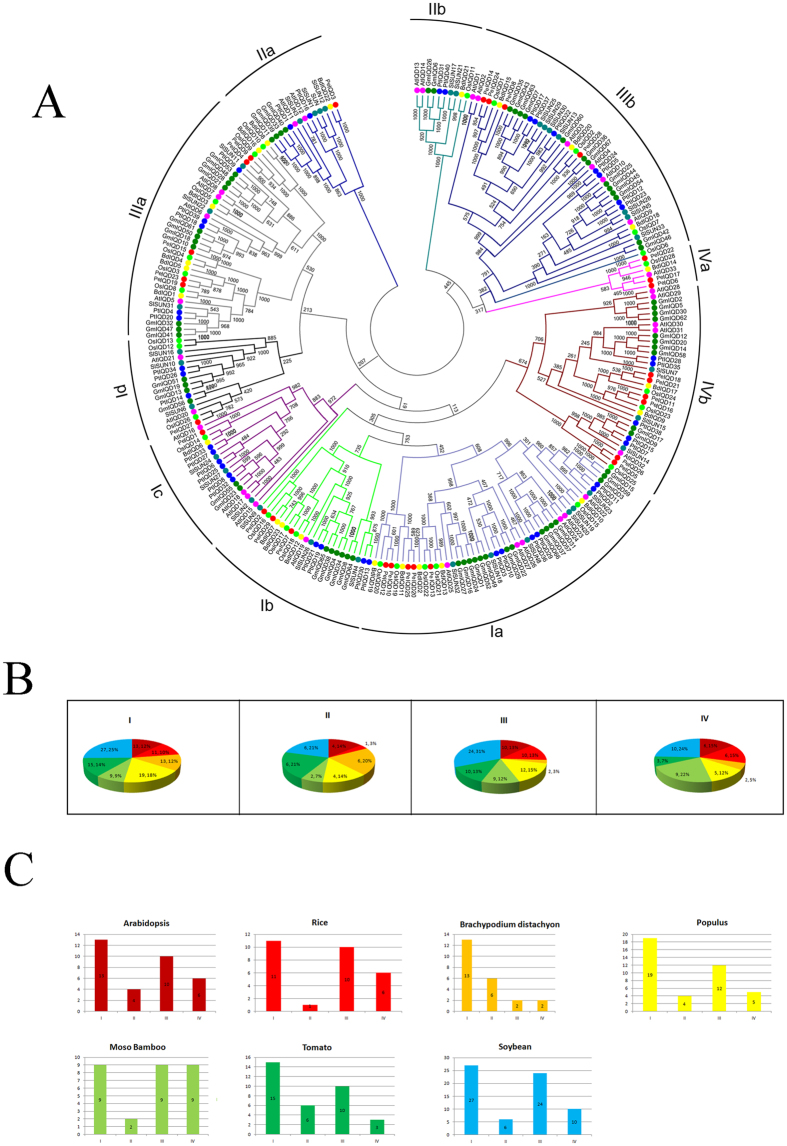
Phylogeny and distribution of IQD protein from seven plant species. (**A**) Phylogenetic tree of IQD proteins from *Arabidopsis*, rice, tomato, *Brachypodium*, soybean, *Populus* and moso bamboo. The tree was generated with Clustal X 2.0 software using the neighbour-joining method. (**B**) Percentage representation of IQD proteins across the seven plant species within each subfamily. Colours correspond to the plant taxa as listed in (**C**). (**C**) Percentage representation of distributions for IQD proteins within each plant species.

To examine the phylogenetic relationship of the IQD domain proteins among the three grass subfamilies – including Pooideae (*Brachypodium distachyon*), Ehrhartoideae (rice), Bambusoideae (*Phyllostachys edulis*) and four dicots (*Arabidopsis thaliana*, soybean, tomato and the woody plant poplar), a rooted tree was constructed from alignments of the full-length IQD protein sequences (Fig. 1A). The phylogenetic tree was constructed using MEGA 6.0 software by employing the Nneighbor-joining (NJ), minimal evolution and maximum parsimony methods. The tree topologies produced by the three algorithms were largely comparable with only minor modifications at interior branches (data not shown). Therefore, only the NJ phylogenetic tree was further analyzed in our study. The NJ phylogenetic tree comprised 226 full-length IQD protein sequences from *Oryza sativa* (28), *Brachypodium distachyon* (23), *Arabidopsis thaliana* (34), *Populus trichocarpa* (40), *Glycine max* (67) and *Solanum lycopersicum* (34)^6^,^12^,^13^,^16^,^17^. The characteristics of the IQD genes–including pI, MW, chromosome location, ORF length and amino acids – are showed in Table S1. As indicated in Fig. 1A, the phylogenetic tree highlighted that the IQD family of proteins could be divided into four well-conserved subfamilies, IQD I-VI, as described previously and with significant statistical support^6^,^12^,^13^. We further examined each of the subfamilies containing the IQD genes. The IQDI subfamily was divided into four clades designated as clade a, b, c and d, which was consistent with the nomenclature in previous studies of *Arabidopsis*, rice and soybean^6^,^12^,^13^. The other three subfamilies were separated into two clades (a and b) (Fig. 1A). The phylogenetic tree revealed that the plant IQD sequence distribution predominates with species bias (Fig. 1B). The largest subfamilies of the plant species were found to contain mostly IQD I genes . In contrast, the IQD II subfamily contained the fewest IQD genes – except in the case of *Brachypodium* and tomato, where IQD IV contained the fewest. It also appeared that more IQD IV genes were present in moso bamboo and soybean IQD IV than in other species. In fact, we found nine and ten IQD IV genes to be present in moso bamboo and soybean, respectively, while only three and two IQD IV genes were present in *Brachypodium* and tomato, respectively. The percentage distribution of the IQD proteins among each subfamily was calculated for all seven species (Fig. 1C). Remarkably, there were fewer IQD genes in each subfamily for monocotyledonous than for dicotyledonous.

### Gene structure and conserved motifs of moso bamboo IQD genes

**Figure 2:**
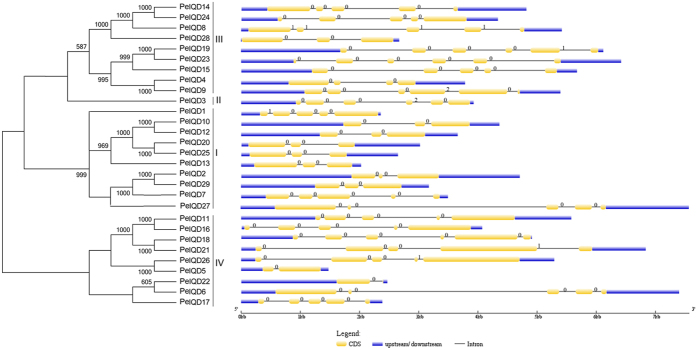
Phylogenetic relationship and intron-exon structure of moso bamboo (*Phyllostachys edulis*) IQD proteins. (**A**) Phylogenetic tree of *PeIQDs* constructed by the neighbour-joining method. Bootstrap values from 1,000 replicates are indicated at each node. The proteins on the tree are divided into four distinct subfamilies. (**B**) Exons and introns are indicated by yellow rectangles and grey lines, respectively. Untranslated regions (UTRs) are indicated by blue lines.

To gain further insights into the structural diversity of moso bamboo IQD genes, we first constructed a separate phylogenetic tree exclusively using the full-length IQD protein sequences of moso bamboo. Moso bamboo proteins were also classified into four independent subfamilies, in good agreement with that described above for the seven plant species (Figs 1A and 2A). It is well known that genetic structural diversity is a possible mechanism for the evolution of multigene families. To gain further insights into the structural diversity of IQD genes, we compared the exon/intron organization in the coding sequences of individual IQD genes in moso bamboo (Fig. 2B). It is interesting to note that we found the intron/exon structure of most sister gene pairs to be conserved, but there are also some differences. For example, the six sister gene pairs (*PeIQD24*/*-14*, *PeIQD19*/*-23*, *PeIQD10*/*-12*, *PeIQD11*/*-16*, *PeIQD20*/*-25* and *PeIQD2*/*-29*) were found to have the same exon/intron number and intron phase. However, their intron lengths showed great variability, ranging from a few tens of bp to −2 kb. In addition, four sister gene pairs showed greater changes in their structural organizations (*PeIQD4*/*-9*, *PeIQD18*/*-21*, *PeIQD26*/*-5*, *PeIQD22*/*-6*) and varying numbers of exons and introns.

**Figure 3:**
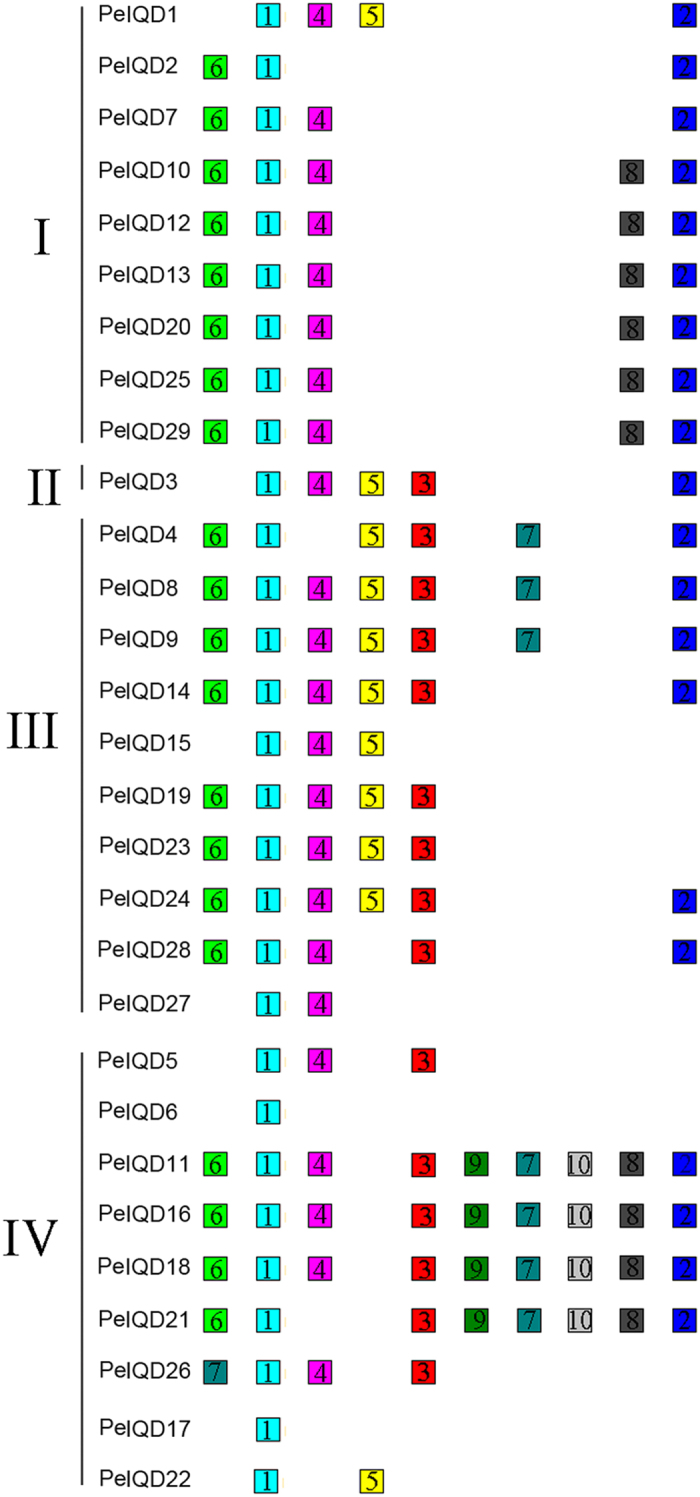
Schematic representation of the 10 conserved motifs in *PeIQD* proteins. Motifs of the *PeIQD* proteins were identified using the online MEME program. Different coloured boxes represent different motifs, with their names in the centre of the boxes. The coloured boxes were ordered manually according to the results of the MEME analysis. The length of each box in the figure does not represent the actual motif size.

We further searched for the conserved motifs in *PeIQD* proteins by MEME program to obtain more insights into the diversity of motif compositions among *PeIQDs*. As shown in Fig. 3, a total of 10 conserved motifs designated as motif 1 to motif 10 were identified. The details of the 10 putative motifs were referred in Table S2. Motif 1 was found to encode the IQ domain, while motif 2 encoded proteins of unknown function (DUF4005). The specific motifs of the other subfamilies have not been functionally annotated. As expected, most of the closely related members had common motif compositions, suggesting functional similarities among IQD proteins within the same subfamily (Fig. 3). The most common motif is motif 1, found in all 29 moso bamboo IQD genes (Fig. 3). To predict calmodulin-binding sites, we searched the Calmodulin Target Database, which provides various structural and biophysical parameters for the 29 *PeIQD* protein sequences. Consecutive strings of amino acid residues with high values of ≥7 indicated the locations of the putative binding sites. The search results showed that all of the predicted *PeIQD* proteins contained at least one CaM-binding site. These CaM-binding sites are described in Table 2.

### Evolutionary patterns and divergence of the IQD gene family in moso bamboo, rice and *Brachypodium*

**Figure 4:**
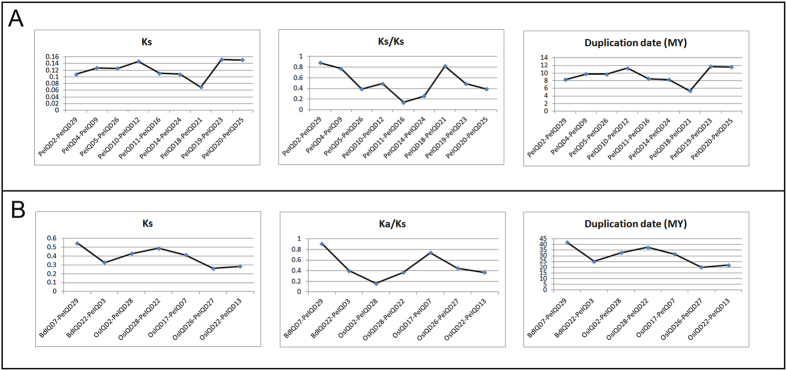
Ks , Ka/Ks value and duplication date (MY) distributions of the IQD genes in the genomes of moso bamboo, rice and *Brachypodium*, and viewed through the frequency distribution of relative Ks and Ka/Ks modes. (**A**) Distribution of Ks, Ka/Ks values and Duplication date (MY) were obtained from orthologous gene-pairs between the moso bamboo and *Brachypodium*, and, between the moso bamboo and rice genomes. (**B**) Distribution of Ks, Ka/Ks values and duplication date (MY) were obtained from paralogous gene-pairs in the moso bamboo genome.

In comparative genomics, the phylogeny-based and bidirectional best-hit methods are popular strategies for identifying possible paralogous or orthologous genes. Using these two methods, we found nine putative paralogous pairs in the moso bamboo genome, five orthologous pairs between *OsIQD* and *PeIQD*, and two orthologous pairs between *BdIQD* and *PeIQD*. All gene-pairs are listed in Table S3. To evaluate the divergence times among these three monocotyledon and gramineous plants, we used a relative Ks measure as a proxy for time. Figure 4(A,B) shows the frequency distributions of the relative Ks values obtained from duplicated and paralogous gene-pairs in the moso bamboo, and from thorthologous pairs between moso bamboo and rice, and, between moso bamboo and *Brachypodium* genomes. The relative Ks distribution peaks around 0.15 in moso bamboo suggested a large-scale event around 12 million years ago (MYA). Peng *et al*. have found that bamboo underwent whole-genome duplication 7–12 MYA^14^, according to analyses of clustered gene families, though our results, indicated that IQD family genes underwent a longer large-scale event. Similarly, the relative Ks distribution peaked at 0.25 for the seven duplicated orthologous gene-pairs, indicating division within the three groups of IQD genes at 20 MYA. A previous study estimated that the divergence time of rice and moso bamboo was 48.6 MYA, and for *Brachypodium* and moso bamboo was 46.9 MYA , and the results of the study by Peng, *et al*.^14^ – combined with those of our own study – revealed that the IQD family have undergone gene evolution after separation of the three progenitors.

To determine the Ka/Ks ratios of different loci in the coding sequences, we performed a sliding-window analysis of the 16 gene pairs (Figure S2). The results showed that the IQD domains have undergone strongly positive selection 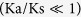, while most loci and regions have undergone moderately or strongly negative selection, as predicted by the overall Ka/Ks ratio.

### Protein interaction analysis of *PeIQDs* with *PeCaM2*

**Figure 5:**
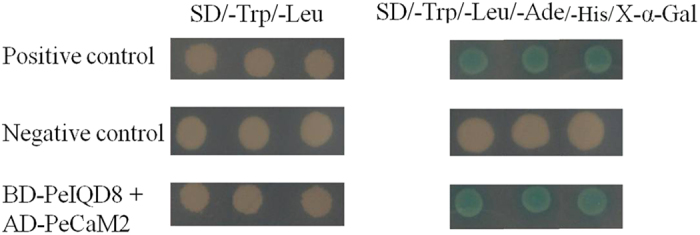
*PeIQDs* interact with *PeCaM2* in yeast. The bait construct of pGBKT7-*PeIQD8* and the prey construct were co-transformed into yeast strain AH109, then examined on SD/-Trp/-Leu and SD/-Trp/-Leu/-Ade/-His/X-α-Gal plates. Positive control, pGBKT7-53 + pGADT7-T; negative control, pGBKT7-Lam + pGADT7-T.

Previous studies have reported that IQD1 and IQD20 can interact with CaM in *Arabidopsis*^6^,^7^. Recently, it was proposed that *ZmIQD15* can interact with CaM *in vitro*^18^. To attest whether this interaction also occurs in moso bamboo, yeast two-hybrid analysis was adopted to examine the interaction between *PeIQDs* and *PeCaM2*. It was verified that expression of BD-PeIQDs and AD-PeCaM2 was not toxic for AH109 yeast cells and that these fusion proteins have no transcriptional activation ability when expressed separately (Figure S3). Our results showed that all the transformants tested were found to grow well on SD/-Leu/-Trp medium. When transferred onto SD/-Trp/-Leu/-Ade/-His/X-α-Gal plates for 5 days, both the positive control and the experimental group turned blue. In contrast, the negative control displayed no α-galactosidase activity (Fig. 5). The results suggested that *PeIQDs* can interact with *PeCaM2 in vitro*.

### Mutations in the IQ motif of *PeIQD8* result in the loss of affinity CaM binding

**Figure 6:**
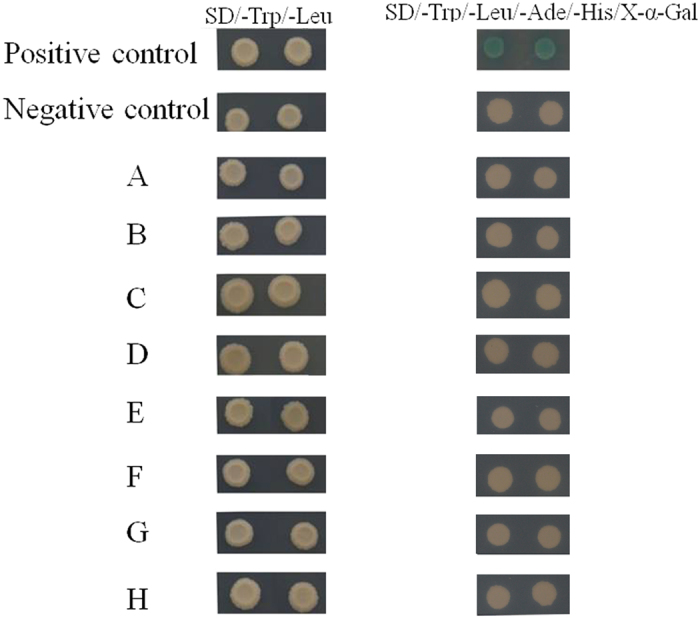
The interaction between *PeCaM2* and *PeIQD8*, *PeIQD8*_*Del112-113*_ or *PeIQD8*_*I112T*_. Positive control, pGBKT7-53 + pGADT7-T; negative control, pGBKT7-Lam + pGADT7-T. (**A**) pGBKT7-PeIQD8 + pGADT7-PeCaM2; (**B**) pGBTKT7- PeIQD8 + pGADT7; (**C**) pGBKT7-PeIQD8_Del112-113_ + pGADT7-PeCaM2; (**D**) PeIQD8_Del112-113_ + pGADT7; (**E**) pGBKT7-PeIQD8_I112T_ + pGADT7-PeCaM2; (**F**) pGBKT7-PeIQD8_I112T_ + pGADT7; (**G**) pGBKT7-Lam + pGADT7-PeCaM2; (**H**) pGBKT7-PeIQD8 + pGADT7-T.

When the recombinant plasmid pGBKT7-PeIQD8_Del112-113_ and pGBKT7-PeIQD8_I112T_ were transformed into the yeast AH109 strain, the self-activation was lost (Figure S3). From Fig. 6, we can clearly see that only the pGBKT7-PeIQD8 and pGADT7-PeCaM2 were co-transformed into the AH109 yeast strain, the selective agar plates (SD/-Trp/-Leu/-Ade/-His/X-α-Gal) turned blue. However, the yeasts that were co-transformed with pGBKT7-PeIQD8_Del112-113_ and pGADT7-PeCaM2 or pGBKT7-PeIQD8_I112T_ and pGADT7-PeCaM2 were not able to grow on the selective medium. The results suggested that the IQ or I in the IQ motif is required for PeIQD8 to combine with CaM2.

### Analysis of the putative promoter regions of the IQD gene family

*Cis*-regulatory elements largely determine the tissue-specific or stress responsive expression patterns of genes^19^, and multi-stimulus responsive genes are closely correlated with *cis*-regulatory elements in the promoter regions^20^,^21^. The function of the IQD gene family has been studied for many species (*Populus*, soybean, maize, and others); such genes have been found to be mainly regulated by drought and MeJA stresses. Two types of *cis*-elements, including the dehydration-responsive element and the wound element were detected in the current study. This observation prompted us to investigate possible stress-responsive *cis*-elements in the promoter regions of the moso bamboo IQD genes by searching the PLACE database. *Cis*-elements located upstream of genes comprising up to 2000 bp have marked effects on binding to target genes^22^,^23^, so the 2000-bp putative promoter regions were used to identify putative stress-responsive *cis*-regulatory elements. The results (Table S5) showed that numerous abiotic stress *cis*-elements – S000176 and S000415 for drought stress, and S000457 for wound stress – were found widely in the promoter regions of the IQD genes in moso bamboo. This clearly showed that IQD gene family respond to abiotic stress and have potential functions in enhancing abiotic stress resistance. For instance, *PeIQD13* possessed up to 22 drought-stress elements (S000415) and *PeIQD9* had up to15 wound-stress elements (S000457). Further analysis of the putative promoter regions of the *PeIQD* gene family will be important in advancing our understanding of the stress tolerance mechanism in moso bamboo.

### Differential expression profiling of *PeIQD* genes

**Figure 7:**
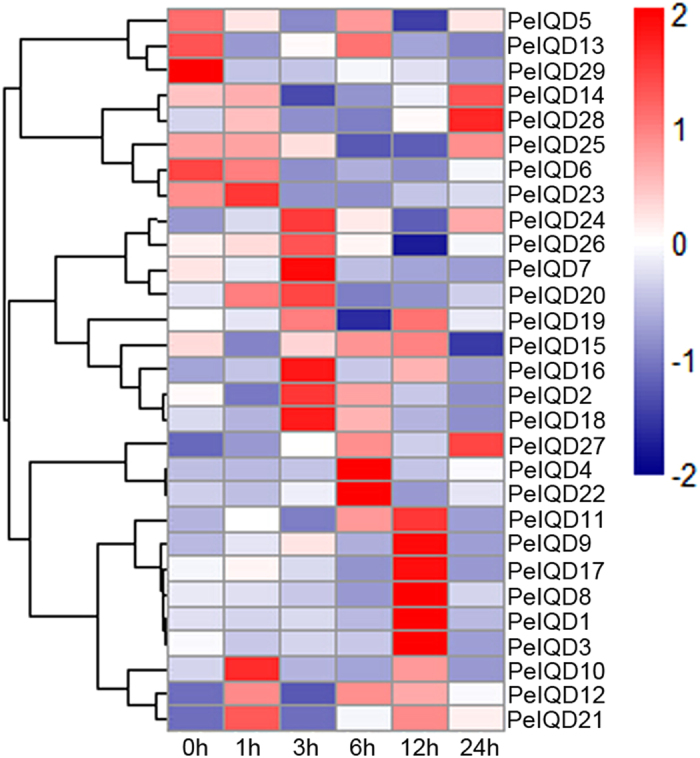
Heat map of the real-time quantitative PCR (qRT-PCR) analysis results of *PeIQD* genes in leaves under drought treatment, with three biological and technical replicates. The scale representing the relative signal intensity values is shown above. Hierarchical clustering was was used in the data analysis.

In plants, quite a few members of the IQD family have been showed to be regulated by insect injury, disease, and drought^6^,^12^,^13^. However, no IQD genes responsive to drought and MeJA stresses have been reported in moso bamboo. We used qRT-PCR to analyze the expression levels of *PeIQD* family genes under drought and MeJA treatments. As shown in Fig. 7, drought (PEG) treatment caused a marked change in the transcription levels of all 29 IQD genes. To clearly realize the expression patterns of the 29 *PeIQD* genes, we further revealed the expression levels of each gene for each time period (Figure S5). Of the 29 genes, 14 (*PeIQD9*, −*10*, −*11*, −*12*, −*14*, −*16*, −*7*, −*20*, −*1*, −*23*, −*24*, −*26*, −*27* and −*28*) showed the highest transcript levels in response to drought (PEG) treatment. Eight genes were expressed specifically during early treatment: *PeIQD10*, −*12*, −*21* and −*23* were highly expressed at 1 h, and *PeIQD16*, −*20*, −*24* and −*26* were highly expressed at 3 h. The expression level of *PeIQD12* and *PeIQD21* at 1 h was 6-fold greater than that at 0 h. *PeIQD16* at 3 h was 13-fold than that at 0 h. In addition, *PeIQD9*, −*11* and −*17* were strongly expressed at 12 h, while *PeIQD14*, −*27* and −*28* were strongly positive expressed at 24 h. In fact, the expression level of *PeIQD9* was enhanced by more than 12-fold.

**Figure 8:**
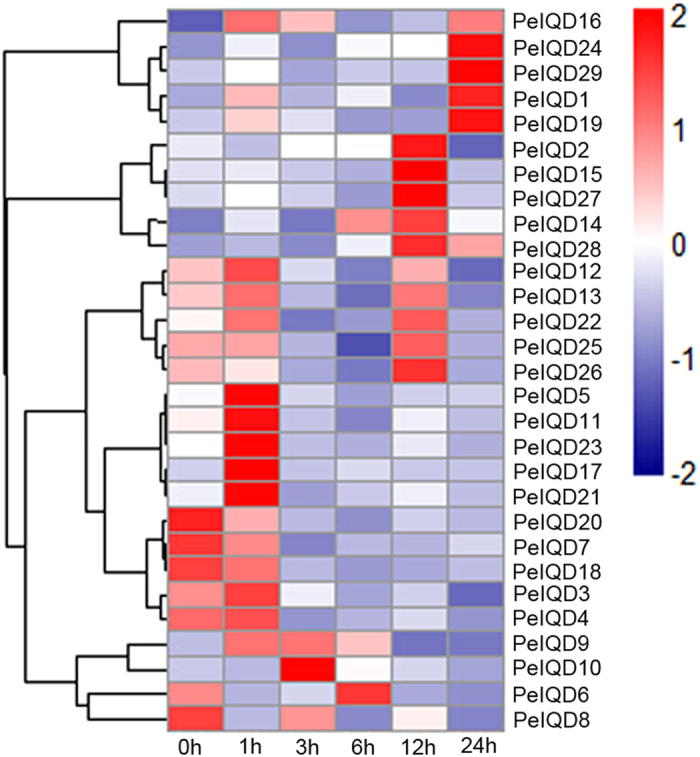
Heat map of the real-time quantitative PCR (qRT-PCR) analysis results of *PeIQD* genes in leaves under MeJA treatment, with three biological and technical replicates. The scale representing the relative signal intensity values is shown above. Hierarchical clustering was used in the data analysis.

From the heat map of the real-time quantitative PCR (qRT-PCR) analysis results for the *PeIQD* genes, we can see that all 29 genes were found to be MeJA responsive, but that some differences were observed among these genes (Fig. 8). Similarly, the expression patterns of 29 *PeIQD* genes under MeJA stress were revealed by qRT-PCR (Figure S6). It can be seen that only nine genes (*PeIQD2*, −*6*, −*7*, −*8*, −*10*, −*18*, −*20*, −*25* and −*26*) have constitutively weak expression levels under MeJA stress. *PeIQD2*, −*6* and −*10* were downregulated during early treatment but upregulated at later time points. For example, *PeIQD10* was highly expressed at 12 h (five-fold). Although 20 genes were upregulated by MeJA treatment, *PeIQD29* was obviously upregulated at all time points. Eleven *PeIQD* genes (*PeIQD1*, −*14*, −*15*, −*16*, *-17*, −*19*, −*21*, −*23*, −*24*, −*27* and −*28*) exhibited major changes in expression (relative expression scale changed from 0 to 5 to 0 to 25). The expression of five genes (*PeIQD10*, −*14*, −*15*, −*27* and −*28*) peaked at 12 h; *PeIQD16* and *PeIQD17* were found to primarily express at 1 h (more than 14-fold and 25-fold, respectively). By contrast, nine genes exhibited minor changes in expression (relative expression scales from 0 to 3 and lower), including *PeIQD2*, −*3*, −*4*, −*5*, −*9*, −*11*, −*12*, −*13* and −*22*.

Together, the qRT-PCR results indicated that 11 genes (*PeIQD1*, −*3*, −*4*, −*5*, −*10*, −*15*, −*19*, *20*, −*22*, −*26* and −*29*) had different patterns under two treatments. For example, *PeIQD10*, *-20* and −*26* were upregulated under drought (polyethylene glycol, PEG) treatment, but downregulated under MeJA stress. *PeIQD9*, −*11*, −*12*, −*14*, −*16*, −*17*, −*21*, −*23*, −*24*, −*25*, −*27* and −*28* were upregulated under two stress treatments, while *PeIQD2*, −*6*, −*7* and −*18* were downregulated under the same treatment, implying that certain *PeIQD* genes play important roles in regulating the responses to drought and MeJA stress. Moreover, while some duplicated genes within a sister pair exhibited similar expression patterns, differential expression patterns between two duplicated genes were observed. For example, under drought (PEG) stress, the highest expression level of *PeIQD4* was observed at 6 h, while that of *PeIQD9* was observed at 12 h. Under MeJA stress treatment, *PeIQD16* exhibited major changes in expression, while *PeIQD11* exhibited only minor changes in expression.

## Discussion

The plant-specific IQD gene family has been comprehensively analyzed in many plants, including *Arabidopsis*, *Oryza sativa*, *Brachypodium*, *Populus*, soybean and tomato, but no IQD genes have been found in algae, suggesting that IQD proteins belong to an ancient family of CaM/CML-binding proteins and developed during the early evolution of land plants. Predicted IQD-like gene in Physcomitrella patens showed that the IQD gene family evolved during the early evolution of land plants 450–700 Mya ago^24^,^25^. In our study, we identified and characterized 29 IQ67 domain-encoding genes in moso bamboo using genome wide analysis, and compared these with 34 *AtIQDs*, 28 *OsIQDs*, 23 *BdIQDs*, 40 *PtIQDs*, 67 *GmIQDs* and 34 *SlIQDs*, indicating that the number of IQD genes in moso bamboo (29) is higher than that in *Oryza sativa* and *Brachypodium distachyon*. but lower than that in dicotyledons, such as *Arabidopsis thaliana*, *Populus trichocarpa*, *Glycine max* and tomato^6^,^12^,^13^,^16^. In these plants, genome sizes vary enormously; for example, the genome size for moso bamboo is 2021 Mb^14^, for *Brachypodium* 300 Mb^17^, for *Arabidopsis* 164 Mb, for rice 441 Mb^26^, for *Populus* 483 Mb^27^, for *Glycine max* 1100 Mb^28^ and for tomato 950 Mb^29^. There are some duplication events may help us to understand the higher number of *IQD* genes within the smaller genome size for *Arabidopsis*, soybean, *Populus* and so on. At least four different large-scale duplication events were shown to have occurred in the *Arabidopsis* genome 100–200 million years ago, and 17% of all genes were shaped in tandem arrays^30^,^31^. The genome duplications of soybean occurred at approximately 59 and 13 million years ago, resulting in a highly duplicated genome with nearly 75% of the genes present in multiple copies^28^. And the *Populus* genome has undergone at least three rounds of whole genome duplications in its evolutionary history^27^. The 226 IQD proteins were found to be divided into four subfamilies, with every subfamily containing both monocotyledons and dicotyledons members, indicating that IQD genes had diversified before the monocot-dicot split. Noticeably, IQD genes with the same functions showed a tendency to be grouped into one subfamily, similar to findings of the previous reports^6^,^12^,^13^,^22^. For instances, subfamily IQDIII encompassed the IQD proteins involved in drought and MeJA stresses (*GmIQDIII* and *PtIQDIII*)^12^,^13^. Moreover, we sought additional evidence – such as gene structure, motif compositions and expression patterns as described above – to support the reliability of the subfamily classification.

In order to research the occurrence of the whole-genome duplication event and the tetraploid origin of bamboo, we investigate the gene collinearity in moso bamboo, rice and *Brachypodium*, as the most recent whole-genome duplication was likely linked to polyploidy events^14^. Recent gene duplication events, which play an important role in the rapid expansion and evolution of gene families, result in many paralogous pairs in different species^32^. And large-scale duplication events are defined as simultaneous duplications of genes. To better explain the patterns of macroevolution, estimates of the evolutionary rates are extremely useful. We estimated the Ks and Ka models of paralogous genes (Pe-Pe) and orthologous genes (Pe-Os and Pe-Bd), than the Ks value was calculated for each gene-pair. Lastly, we use the formula (T = Ks/2λ) to calculate the approximate date of the duplication event, assuming a λ value of synonymous substitution of 6.5 × 10^–9^ for moso bamboo, rice and *Brachypodium*^14^,^33^,^34^. We estimated the large-scale event occurred approximately was 5–12 MYA in moso bamboo, and the divergence times for orthologous genes (Pe-Os and Pe-Bd) to be 20–35 MYA and 25–40 MYA . Peng *et al*. estimated that the divergence time for moso bamboo and rice was 48.6 MYA, and for moso bamboo and *Brachypodium* was 46.9 MYA^14^. According to the ratio of nonsynonymous to synonymous substitutions (Ka/Ks), the history of selection acting on coding sequences can be measured. In general, Ka/Ks ratio greater than 1, less than 1 and equal to 1 represents positive selection, negative or stabilizing selection and neutral selection, respectively^32^,^35^. In the current study, the Ka/Ks ratios were less than 1 in moso bamboo, indicating that the nine gene pairs experienced purifying selection during the process of evolution. The Ka/Ks ratios were less than 0.9 for Pe-Os and Pe-Bd, indicating that the seven gene pairs experienced negative or purifying selection during the process of evolution.

The characteristic of the IQ67 domain is remarkable: it is characterized by a unique and repetitive arrangement of three different calmodulin recruitment motifs, known as the IQ, 1–5–10, and 1–8–14 motifs. In addition, these motifs contain some basic and hydrophobic amino acid residues^6^,^36^. All predicted *PeIQD* proteins have a typical “IQ calmodulin-binding motif” domain (PF00612) which is a major calcium (Ca^2+^) regulator (Fig. 3). Additional*, PeIQD* proteins are constructed by multiple sequence alignment of their IQ domains, which span approx 67 amino acids (Figure S1). Previous studies have revealed that at least five *Arabidopsis* protein families play a role in the calcium signalling pathway because they contain common IQ motifs, such as the cyclic nucleotide gated channels (CNGC) family, the IQ-Motif (IQM) family, the CaM-binding transcriptional activator (CAMTA) family, the myosin family, and the IQD family^37^,^38^,^39^, which contain one, one, two, five and up to three IQ motifs, respectively. In our study, the *PeIQD* proteins contained three IQ motifs. Furthermore, the presence of possible CaM binding sites was predicted by using the Calmodulin Target Database for all the *PeIQD* proteins; all of the predicted *PeIQD* proteins were found to contain at least one CaM-binding site (Table 2), suggesting that these proteins are a typical class of CaM targets. We also found that every *PeIQD* protein contains CaMBDs (CaM binding domains) of different primary structures, indicating that CaMBD is not a conserved domain. In the IQD family, some proteins have function in calcium signaling pathway were testified. *AtIQD1* can control glucosinolate accumulation through binding to CaM in a Ca^2+^-dependent fashion under biotic stresses^8^. *AtIQD20* also interacts with CaM in a Ca^2+^-independent manner^6^. *AtIQD26* and *AtCaM2* (AT2G27030) can be shown to interact by means of the yeast two-hybrid experiment^36^. It was noteworthy that the amino acid sequences for *AtCaM2* (AT2G27030) and PH01000202G0880 were 97.32% similarity (Figure S4). We used the InterproScan program to examine that PH01000202G0880 has the CaM domain and named it *PeCaM2*. Next, we used yeast two-hybrid analysis to detect the interaction between CaM and *PeIQDs*, and we randomly selected *PeIQD8* to be tested. Our results showed that *PeIQD8* can interact with *PeCaM2*, which provides further favourable evidence that *PeIQDs* may perform their functions by interacting with CaM. Zhou *et al*. proved that *AtIQM1* (IQ motif-containing protein) can bind with CaM5 via its IQ-motif^40^. To investigate the important role of the IQ motif in the interaction between IQD proteins and CaMs, we designed two mutant proteins (*PeIQD8*_*Del112*-*113*_ and *PeIQD8*_*I112T*_) to verify the binding characteristics of *PeIQD8* with CaM2. Finally, we found that the two mutant proteins could not interact with *PeCaM2*. These results implied that the IQ motif is the key structural domain and that the I in the IQ motif is the key amino acid residue determining this binding activity. Furthermore, the interacting partners within IQD proteins promote the reconstruction of signalling pathways that involve IQD proteins.

In *Arabidopsis*, Jasmonic acid (JA) treatment leads to elevating levels of specific glucosinolate^41^,^42^, and glucosinolates are a small but diverse class of defense related secondary metabolites, which play an important roles in plant defence^43^. The overexpression of *AtIQD1* causes the accumulation of glucosinolates and increases resistance against herbivory by augmenting and fine-tuning glucosinolate accumulation^7^. In addition, Feng *et al*. verify that 24 soybean IQD III genes are regulated by MeJA stress^12^. Similarly, the expression of 12 selected *PeIQD* members are regulated by MeJA. The IQD genes are also regulated by drought treatment. For example, the *PtIQDIII* members and *ZmIQD* genes have been testified^12^,^18^. Base on the phylogenetic analysis of IQD members, we used further qRT-PCR to investigate the expression patterns of *PeIQD* family genes under MeJA and drought treatments. The results demonstrated that the *PeIQDs* genes were either increased or repressed under the PEG and MeJA treatments, and we speculated that moso bamboo IQD genes might have the similar biological function in defence to insect herbivory and drought stress. By considering all of the results for abiotic stresses, we deduced that most genes within the same subclass of the phylogenetic tree showed different expression patterns. For example, three pairs of duplicated genes (*PeQD5*-*PeIQD26*, *PeIQD18*-*PeIQD21* and *PeIQD19*-*PeIQD23*) showed the different expression patterns under drought (PEG) stress; similarly, six pairs of duplicated genes (*PeIQD10*-*PeIQD12*, *PeIQD20*-*PeIQD25*, *PeIQD2*-*PeIQD29*, *PeIQD18*-*PeIQD21*, *PeIQD5*-*PeIQD26* and *PeIQD6*-*PeIQD22*) showed similar expression patterns under MeJA stress. This is because the regulatory sequences that responded to the stress conditions had diverged dramatically along with the evolution of each gene after duplication. In contrast, some pairs of duplicated genes expressed similar patterns, indicating that these duplicated genes in the same subclass share a high sequence similarity. Here, the expression patterns were similar, revealing that the regulatory sequences that responded to the stress conditions diverged to a much lesser extent the evolution of each gene after duplication. The new information obtained in this study may aid in the selection of appropriate candidate genes for further functional characterization.

## Materials and Methods

### Identification of IQD family genes in the moso bamboo genome

The conserved IQ domain (PF00612) was originally applied as a probe to search the National Center for Gene Research database (http://www.ncgr.ac.cn/bamboo)^14^. All redundant sequences were discarded from further analysis based on cluster W alignment results^44^, sequence identification numbers and chromosome location. Furthermore, to verify the reliability of the initial results, all non-redundant candidate IQD sequences were analyzed to confirm the presence of the conserved IQ domain using the InterproScan program^45^. A total of 33 *Arabidopsis thaliana*, 23 *Brachypodium distachyon*, 40 *Populus* and 67 *Glycine max* IQD protein sequences were downloaded from Phytozome v10.3 (http://www. phytozome.net/), and 34 tomato IQD protein sequences were retrieved from the tomato WGS chromosomes (2.40; SL2.40) (SGN http://solgenomics.net). Finally, 27 rice IQD protein sequences were obtained from the TAIR database (http://rice. plantbiology.msu.edu). The accession numbers of the published IQD proteins for *Arabidopsis thaliana*, rice, tomato, *Brachypodium distachyon*, *Glycine max* and *Populus* are listed in Table S1. Moso bamboo IQD gene information, including the number of amino acids, ORF lengths and chromosome locations were obtained from the National Center for Gene Research (http://www.ncgr.ac.cn/bamboo). Physicochemical parameters including the molecular weight (MW) and isoelectric point (pI) of each gene product were calculated using compute the pI/Mw tool from ExPASy (http://www.expasy.org/tools/) and the parameter (resolution) was set to average^46^. We used the Gene Structures Display Server (GSDS) (http://gsds.cbi.pku.edu.cn/) to illustrate the exon/intron structure for individual IQD genes by comparing their cDNAs and the corresponding genomic DNA sequences^47^.

### Identification of the conserved motifs and putative calmodulin-binding sites

The conserved motifs were analyzed using MEME version 4.11.1^48^,^49^. The identified protein motifs were further annotated with ScanProsite^50^. All IQD protein sequences were examined against the Calmodulin Target Database (http://calcium.uhnres.utoronto.ca/ctdb/ctdb/home.html) to predict putative calmodulin-binding sites^51^.

### Evolutionary patterns and divergence of the IQD gene family in moso bamboo, rice and *Brachypodium* analysis

Pairwise alignment of IQD gene encoding sequences of the orthologous and paralogous pairs was performed using ClustalX 2.11 software. Paralogous IQD gene pairs in moso bamboo were identified on the basis of alignment results. The criteria described in previous studies were adopted^52^,^53^: the shorter sequences covers over 70% of the longer sequence after alignment and the minimum identity of aligned regions is 70%. To identify putative orthologues between two species (A and B), each sequence from species A was searched against all sequences from species B using the BLASTN tool. Additionally, each sequence from species B was searched against all sequences from species A. The two sequences were defined as orthologues if each of them was the best hit of the other, and if more than 300 bp of the two sequences aligned. In addition, to further analyze gene duplication events, the synonymous substitution rate (Ks) and nonsynonymous substitution rate (Ka) were calculated using the software DnaSp^54^,^55^. The date of duplication events was subsequently estimated according to the equation T = Ks/2λ. The approximate value for clock-like rate was 6.5 synonymous substitutions per 10^−9^ years for moso bamboo, rice and *Brachypodium distachyon*^14^,^33^. A sliding window analysis of Ka/Ks ratios was performed with the following parameters: window size, 150 bp; step size, 9 bp.

### Yeast two-hybrid assay

The Matchmaker GAL4 two-hybrid system (Clontech, Palo Alto, CA) was used to test the interaction between *PeIQDs* and CaM. The full length of *PeIQD8* and *PeCaM2* (PH01000202G0880) cDNAs (Table S4) were cloned into the pGBKT7 bait vector and pGADT7 prey vector, respectively. Next, they were co-transformed into yeast strain AH109. The transformed yeast cells were selected on SD/-Trp/-Leu and SD/-Trp/-Leu/-Ade/-His/X-α-Gal plates at 30 °C for 3–5 days to determine the protein-protein interaction. We used pGBKT7-53 and pGADT7-T as a positive control, while the co-transformants with pGBKT7-Lam and pGADT7-T were used as negative controls in the yeast clone experiments.

### Effects of mutations in the IQ motif of *PeIQD8* on its CaM binding capacity

To confirm the effects of mutations of key amino acid residues in the IQ motif of *PeIQD8* on its CaM binding capacity, the IQ was deleted or the I was mutated to T using mutagenesis technology *in vitro*. Two pairs of primers were designed to amplify the IQD8_Del112-113_ (deletion of IQ112–113 in the *PeIQD8* ORF) and IQD8_I112T_ (where I112 was mutated to T112), using the sequencing-confirmed plasmid pEASY-T1 Simple-*PeIQD8* as a template (Table S6). The PCR products were subsequently treated with Dpn I (TaKaRa, Japan) to eliminate methylation. After verification by sequencing, the IQD8_Del112-113_ and IQD8_I112T_ fragments were inserted into *KpnI* and *XbaI*-digested pGBKT7 vector. Finally, the CaM binding capacity of the mutant proteins was analyzed via a yeast two-hybrid assay, as described above.

### Analysis of the putative promoter regions of the IQD gene family in moso bamboo

The 2000-bp upstream sequences of the transcriptional start site of the *PeIQDs* were chosen to identify the *cis*-elements in the putative promoter regions. The PLACE website (http://www.dna.affrc.go.jp/PLACE/signalscan.html) was adopted to identify putative *cis*–elements among the promoter sequences^56^.

### Plant material and growth conditions

Moso bamboo seeds were collected from Guilin, Guang Xi Province, China. The seeds from individual plants were germinated on sterile filter papers in culture dishes, while keeping the filter papers moist and in darkness at 25 °C. The seedlings were transferred to plastic pots containing vermiculite and grown in an illuminated incubator with 16/8 h of light/dark at 25/18 °C and 80% humidity, and watered with Hoagland nutrient solution every week. Plants were cultivated for three months. For the stress treatments, young leaves were sprayed with either 100 uM MeJA or 20% PEG-6000 solution and sampled at five time points (1, 3, 6, 12 and 24 h) after treatment. All samples were immediately frozen in liquid nitrogen and stored at −80 °C for RNA extraction after collection. The untreated samples were used as the control (0 h).

### RNA isolation and qRT-PCR

The total RNA from young leaf was extracted using TRIzol reagent (Invitrogen, Ca, USA) according to the manufacturer’s instructions. The total RNA was extracted from frozen samples using an RNAprep Pure Plant Kit (Tiangen) according to the manufacturer’s instructions. The first-strand cDNA was then synthesized using a PrimeScriptTM RT Reagent Kit (TaKaRa). Gene-specific primers were designed using Primer Express 3.0 and Tonoplast intrinsic protein 41 gene (TIP41) was used as the reference gene^57^. Real-time PCR was performed on an ABI 7300 Real-Time system (Applied Biosystems). Each reaction was carried out in a final volume of 20 ul containing 12.5 μl of SYBR Green Master Mix reagent (Applied Biosystems), 1.5 μl of cDNA sample, and 1μl gene-specific primers. Each primer pair of *PeIQD* genes was listed in Table S6. The qPCR reaction conditions were as follows: 50 °C for 2 min, 95 °C for 10 min, 40 cycles of 95 °C for 15 s, and annealing at 55–60 °C for 30 s. A melting curve was generated to analyze the specificity of the reactions, and three biological replicates were made for each biological replicate. The relative expression level was calculated as 2^−ΔΔCT^ [ΔC_T_ = C_T_, _Target_ − C_T_, _CYP2_. ΔΔCT = ΔC_T_, _treatment_ − ΔC_T_, _CK_ (0 h)].The relative expression level [2^−ΔΔCT, CK (0 h)^] in the control plants (without treatment) was normalized to 1 as described previously^58^.

## Conclusions

In the current study, we systematically identified and characterized by bioinformatics the plant-specific IQD gene family of putative calmodulin target proteins in the only Bambusoideae plant, moso bamboo (*Phyllostachys edulis*). We explored 29 IQD genes in the moso bamboo genome and explored their expression profiles under drought (PEG) and MeJA conditions, which were investigated using quantitative real-time PCR (qRT-PCR). The qRT-PCR results elucidated the precise role of the individual *PeIQD* gene. Yeast two-hybrid analysis revealed that *PeIQD8* can interact with *PeCaM2*. This study presents a thorough overview of the moso bamboo IQD gene family and provides a new perspective on the evolution of this gene family. These data will provide an insight into further understanding of the functions of IQD members and their roles bamboo in moso bamboo growth and development.

## Additional Information

**How to cite this article**: Wu, M. *et al*. Genome-wide identification and expression analysis of the IQD gene family in moso bamboo (*Phyllostachys edulis*). *Sci. Rep*. **6**, 24520; doi: 10.1038/srep24520 (2016).

## Supplementary Material

Supplementary Information

## Figures and Tables

**Table 1:** Detailed information about the IQD proteins in moso bamboo.

Gene name	Sequence ID	Chr	Location	ORF length	PI	Mol. Wt. (Da)	Size (aa)	Exons
PeIQD1	PH01000003G1440	PH01000003	967133–969487 (+stand)	1581	10.97	56786.68	526	5
PeIQD2	PH01000025G1940	PH01000025	1440447–1445151 (+stand)	1164	10.12	40499.31	387	3
PeIQD3	PH01000047G0550	PH01000047	413725–417652 (+stand)	1284	10.21	47172.98	427	6
PeIQD4	PH01000048G0670	PH01000048	465999–469782 (−stand)	1215	10.8	44104.8	404	4
PeIQD5	PH01000057G1170	PH01000057	819186–824476 (+stand)	2574	5.02	94051.68	857	2
PeIQD6	PH01000062G1950	PH01000062	1326243–1328709 (+stand)	573	7.97	20417.05	190	5
PeIQD7	PH01000208G0940	PH01000208	774898–778391 (+stand)	1359	10.03	49944.89	452	5
PeIQD8	PH01000232G0210	PH01000232	100923–106341 (−stand)	1410	10.31	51361.34	469	5
PeIQD9	PH01000241G1120	PH01000241	727425–732816 (+stand)	2100	11.12	75521.94	699	6
PeIQD10	PH01000252G0640	PH01000252	542193–546553 (+stand)	1131	10.59	42409.3	376	3
PeIQD11	PH01000271G0580	PH01000271	407684–415256 (−stand)	1665	9.63	61112.7	554	5
PeIQD12	PH01000284G0220	PH01000284	125209–128867 (+stand)	1122	10.29	41965.83	373	3
PeIQD13	PH01000358G0650	PH01000358	525937–527963 (−stand)	1377	10.58	49279.03	458	3
PeIQD14	PH01000428G0560	PH01000428	373345–378158 (−stand)	1422	10.09	52057.07	473	5
PeIQD15	PH01000458G0650	PH01000458	459032–464703 (+stand)	1050	7.23	38832.77	349	5
PeIQD16	PH01000502G0350	PH01000502	306474–312052 (+stand)	1782	9.52	65347.29	593	5
PeIQD17	PH01000742G0510	PH01000742	312291–319680 (+stand)	2823	8.99	105765.41	940	5
PeIQD18	PH01001210G0100	PH01001210	52102-56165 (+stand)	1701	9.75	62065.3	566	6
PeIQD19	PH01001453G0160	PH01001453	91365–97487 (+stand)	1320	9.59	48644.97	439	6
PeIQD20	PH01001731G0460	PH01001473	186745–189760 (−stand)	1074	10.52	39918.54	357	3
PeIQD21	PH01001473G0280	PH01001731	279500–284408 (+stand)	1824	9.56	67194.21	607	5
PeIQD22	PH01002085G0100	PH01002085	37695–40080 (−stand)	873	5.16	32027.29	290	1
PeIQD23	PH01002634G0120	PH01002634	70363–76783 (−stand)	1023	9.3	38307.17	340	6
PeIQD24	PH01003208G0200	PH01003208	131125–135457 (+stand)	1416	10.05	51559.67	471	5
PeIQD25	PH01003304G0090	PH01003304	40750–43396 (−stand)	1083	10.35	40194.03	360	3
PeIQD26	PH01003925G0040	PH01003925	40308-47140 (+stand)	2769	5.1	101962.98	922	5
PeIQD27	PH01004272G0030	PH01004272	8090–9565 (−stand)	894	10.77	31984.13	297	5
PeIQD28	PH01004403G0090	PH01004403	68879–71552 (−stand)	1461	10.5	54111.89	486	3
PeIQD29	PH01004470G0050	PH01004470	15995–19170 (+stand)	1305	10.38	46928.34	434	3

**Table 2:** Predicted calmodulin binding sites in moso bamboo IQD proteins.

Group	Name	Predicted calmoduin binding sequence
I	PeIQD1	3-KGGTSWLTAVKRAFRSP 145-ALKGLVKLQALVRGHNV 261-QTRKDAALKRERALSYAF
	PeIQD2	130-SAIKIQSAFRSYLARKALCA
	PeIQD7	426-RIRKRIWEGGICRIQS
	PeIQD10	92-VVIQKSFRGYLARK
	PeIQD12	9-KKLLTGRKGGHKGLK
	PeIQD13	183-ALVRAQAAIRAARSR
	PeIQD20	8-AAVMIQKAFRGYLARKALRA 103-SLVKLQALVRGYLVRKQAVT
	PeIQD25	85-SAVMIQ KAFKGYLARKALRA 107-SLVKLQALVRGYLVRKQAAT
	PeIQD27	143-GNAKLGRR
	PeIQD29	151-VKMQALVRGHLVRRQAS 172- MQALVAAQNRARAARLR
II	PeIQD3	151-QAVRRQTAATLRGLESLVKIQ
III	PeIQD4	38-SGGQRGAAAGNASA
	PeIQD8	215-QEAGIRRERALAYAF
	PeIQD9	466-SFLLSLMRAAAS
	PeIQD14	216-EAAIR RERALAYAFS
	PeIQD15	165-FRAFLARRARRALKGL
	PeIQD19	156-ARVRARQVRVTLE
	PeIQD23	6-SKWIKSLIGIRKQEKG 125-IVKLQALVRGHIVRKQTA 151-LVRAQARVRARQVRVAL
	PeIQD24	213-QGAAIRRERSLAYAF
	PeIQD28	191-RGLVRLKSLVDGNTVKR
IV	PeIQD5	248-KLQAVIRGHLVRRQAAESLQ
	PeIQD6	118-AVREARRAVTRRVVGLQE
	PeIQD11	166-FQALVRGRNVRLS
	PeIQD16	194-FQALVRGRNVRLSS
	PeIQD17	598-EYHNLKKAISIL 655-RFVRMRRSAIVIQQAVR
	PeIQD18	16-QALVRGRNVRLSS
	PeIQD21	145-LVRRQSVSTLRATLLIVKFQALV 195-KSS
	PeIQD22	227-ERALAYAFSQKL
	PeIQD26	259-KLQAVIRGHLVRRQAAESLQ 893-SNRGTFIYLLAQWRIC

Predicted calmodulin binding sites obtained from the Calmodulin Target Database are shown for strings of amino acid residues with a score of at least 7. Residues with the highest score (9) are highlighted in bold. Numbers before strings indicate the location of the first amino acid residues of the strings in moso bamboo IQD protein sequences.
