# Supplementary material for: Genome-wide identification and expression analysis of the IQD gene family in moso bamboo (Phyllostachys edulis)
Source: Sci Rep. 2016 Apr 20;6:24520. doi: 10.1038/srep24520 (PMC4837358; doi:10.1038/srep24520)
Supplement: Supplementary Information [file srep24520-s1.pdf]

Genome-wide identification and expression analysis of the IQD gene family in moso bamboo (*Phyllostachys edulis*)

Min Wu[1,2], Yuan Li[2], Danmei Chen[2], Huanlong Liu[2], Dongyue Zhu[2], Yan Xiang[2]*

1 Key Laboratory of Crop Biology of Anhui Province, School of Life Sciences, Anhui Agricultural University, Hefei 230036, China

2 Laboratory of Modern Biotechnology, School of Forestry and Landscape Architecture, Anhui Agricultural University, Hefei 230036, China

*Corresponding Author

Yan Xiang

Laboratory of Modern Biotechnology, School of Forestry and Landscape Architecture, Anhui Agricultural University, Hefei 230036, China

Phone: 0551-65781786

Email: xiangyanahau@sina.com

Fig. S1. Amino acid sequence alignments of IQ67 domains in moso bamboo (*Phyllostachys edulis*) IQD protein sequences. The multiple alignment results indicate the highly conserved IQD domains among the 29 identified moso bamboo IQD protein sequences. The shading of the alignment presents residues (white text) of the IQ motifs (brown), the 1-5-10 motifs (dark blue) and the 1-8-14 motifs (yellow).

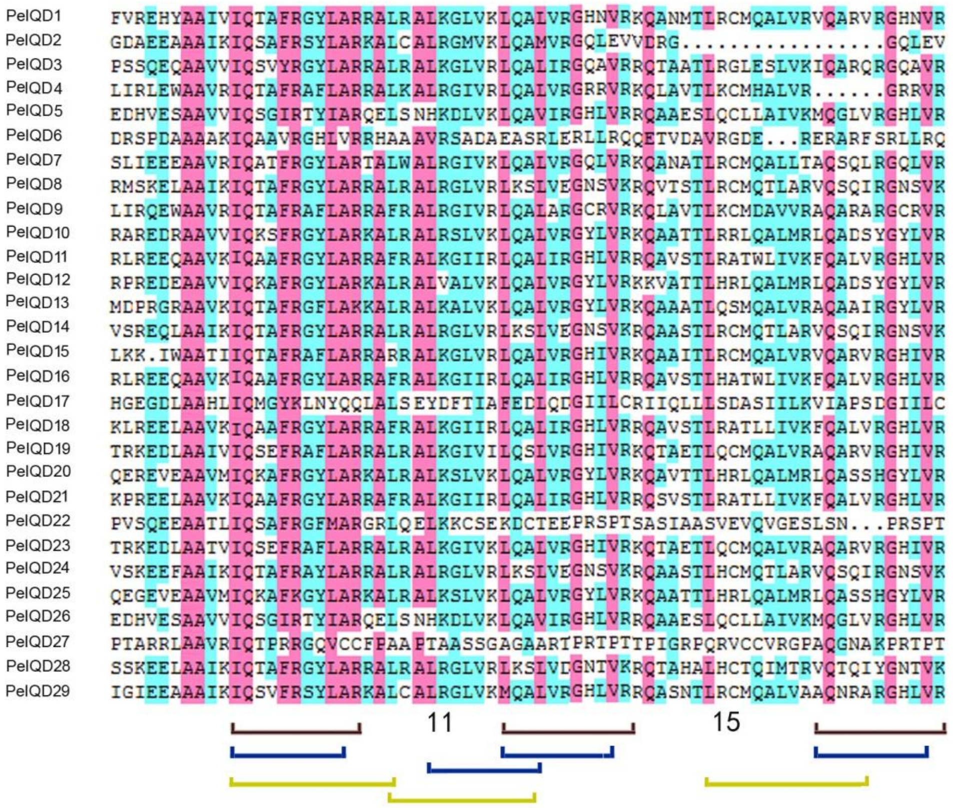

Fig. S2. Sliding window plots of representative duplicated IQD genes in moso bamboo and between moso bamboo and rice, and, between moso bamboo and *Brachypodium*. As shown in the key, dark grey blocks indicate the positions of the IQD domain. The window size was 150 bp, and the step size 9 bp.

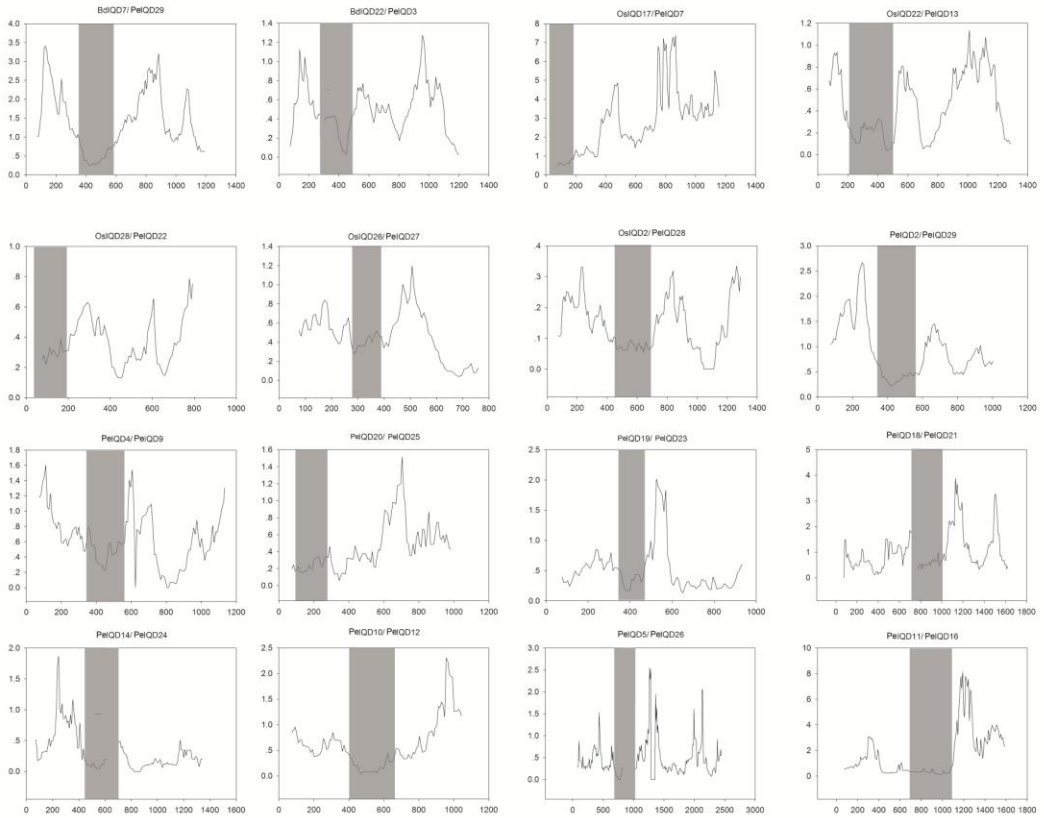

Fig. S3. Self-activation of bait proteins in yeast. Positive control, pGBKT7-53+ pGADT7-T; negative control, pGBKT7; experimental groups, pGBKT7-PeIQD8, pGBKT7-PeCaM2, pGBKT7-PeIQD8$_{Del112-113}$, pGBKT7 PeIQD8$_{I112T}$.

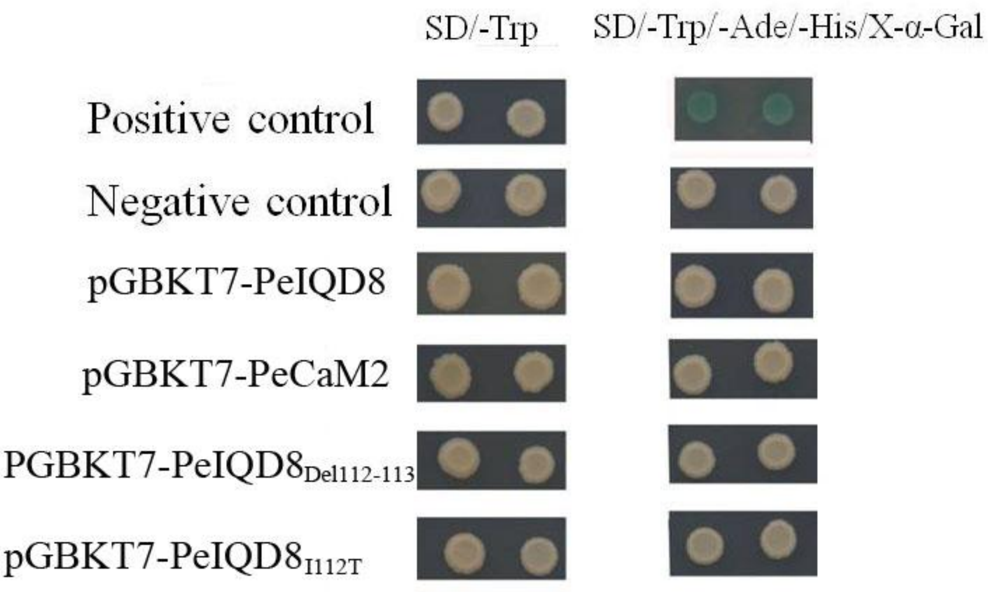

Fig. S4. Amino acid sequence alignments of PeCaM2 and AtCaM2. The multiple alignment results indicate the high similarity of the *PeCaM2* and *AtCaM2* protein sequences.

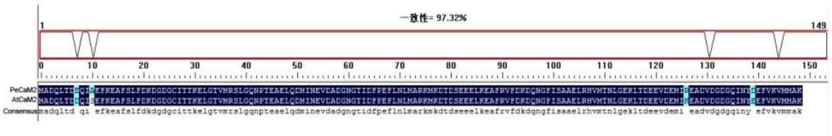

Fig. S5. Expression patterns of 29 *PeIQD* genes under drought (PEG) stress, as revealed by qRT-PCR. The Y-axis indicates the relative expression levels. The X-axis indicates the time courses of stress treatments. Error bars, 6 ±SE.

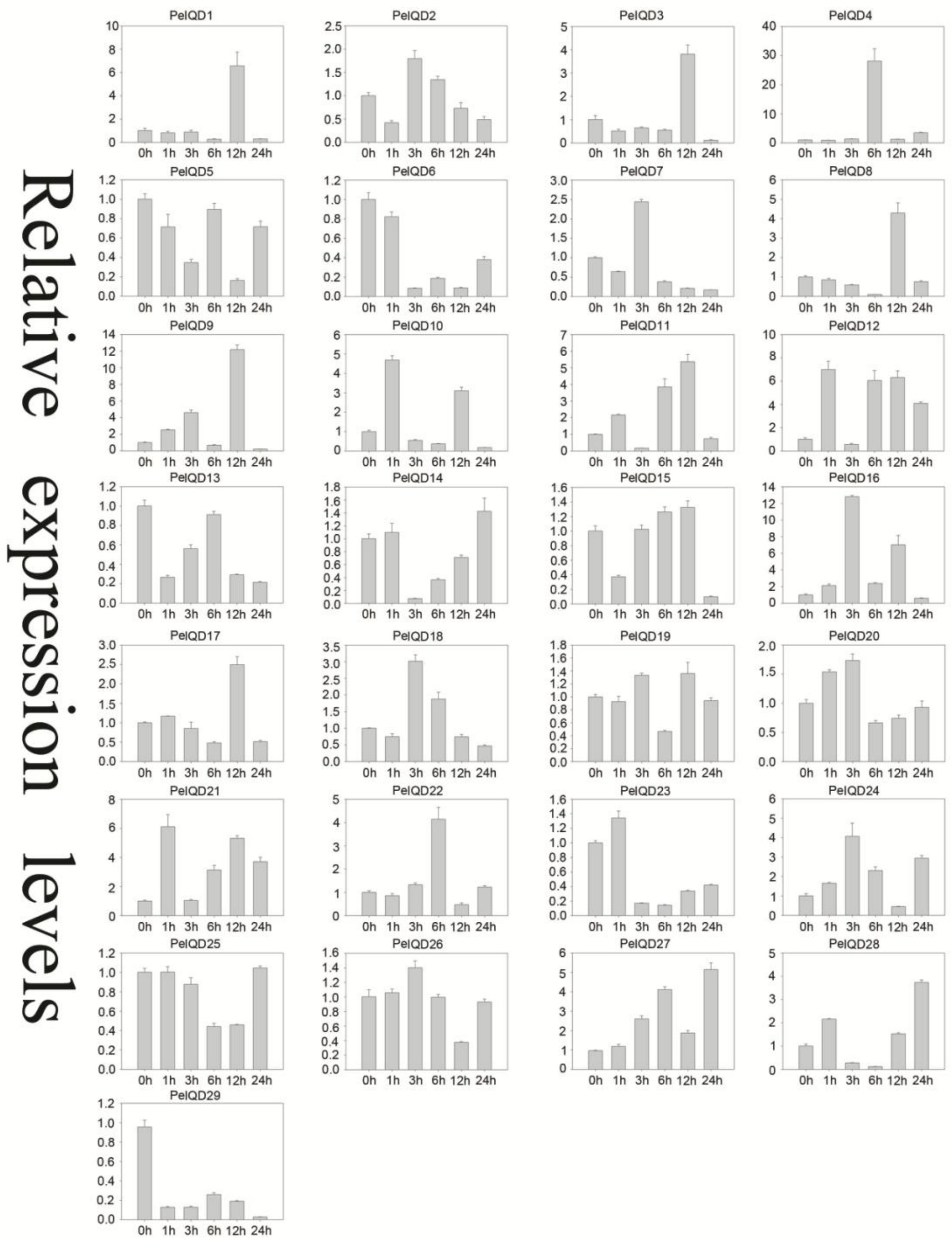

Fig. S6. Expression patterns of 29 *PeIQD* genes under MeJA stress, as revealed by qRT-PCR. The Y-axis indicates the relative expression levels. The X-axis indicates the time courses of stress treatments. Error bars, 6±SE.

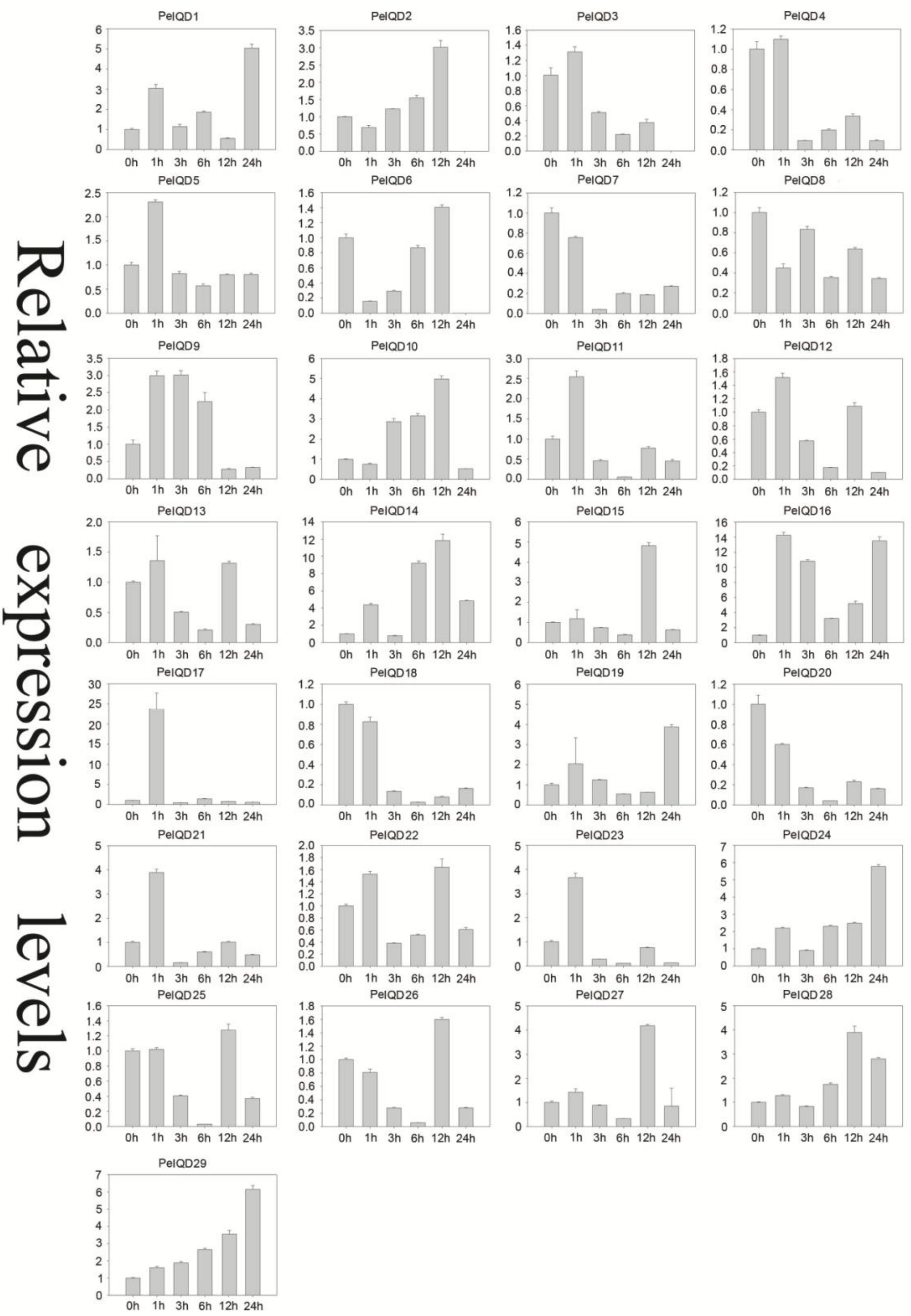

Table S1. Detailed information about IQD proteins in *Arabidopsis*, rice, *Brachypodium*, *Populus*, tomato and soybean.

Gene name	Sequence ID	Location	Chr	ORF length(bp)	Size(aa)	WoLF PSORT
Arabidopsis						
AtIQD1	AT3G09710.1	2976605-2979389	3	1365	454	N
AtIQD2	AT5G03040.1	710102-712788	5	1386	461	N
AtIQD3	AT3G52290.1	19393819-19396403	3	1293	430	?
AtIQD4	AT2G26410.1	11234651-11236936	2	1584	527	?
AtIQD5	AT3G22190.1	7831280-7833904	3	1203	400	N
AtIQD6	AT2G26180.1	11143290-11145103	2	1251	416	N
AtIQD7	AT1G17480.1	6006461-6008266	1	1113	370	?
AtIQD8	AT1G72670.1	27356683-27358853	1	1245	414	N
AtIQD9	AT2G33990.1	14360276-14362241	2	750	249	N
AtIQD10	AT3G15050.1	5066879-5067960	3	780	259	?
AtIQD11	AT5G13460.1	4315759-4318360	5	1332	443	N
AtIQD12	AT5G03960.1	1068260-1069978	5	1212	403	?
AtIQD13	AT3G59690.1	22048754-2205167	3	1854	617	?
AtIQD14	AT2G43680.2	18108349-18111831	2	2007	668	?
AtIQD15	AT3G49380.1	18311909-18313490	3	1059	352	N
AtIQD16	AT4G10640.1	6571899-6574427	4	1272	423	N
AtIQD17	AT4G00820.1	349116-351550	4	1605	534	?
AtIQD18	AT1G01110.2	52239-54692	1	1584	527	N
AtIQD19	AT4G14750.1	8470381-8472187	4	1164	387	?
AtIQD20	AT3G51380.1	19074036-19074426	3	312	103	M
AtIQD21	AT3G49260.1	18262399-18265979	3	1416	471	N
AtIQD22	AT4G23060.1	12087203-12090575	4	1632	543	?
AtIQD23	AT5G62070.1	24929919-24932576	5	1212	403	?
AtIQD24	AT5G07240.1	2271919-2274288	5	1206	401	?
AtIQD25	AT4G29150.1	14378767-14380511	4	1152	383	?
AtIQD26	AT3G16490.1	5603900-5605697	3	1197	398	?
AtIQD27	AT1G51960.1	19310355-19311683	1	1056	351	?
AtIQD28	AT1G14380.1	4918010..4921596	1	1995	664	N
AtIQD29	AT2G02790.1	787915-790946	2	1911	636	N
AtIQD30	AT1G18840.1	6500848-6504413	1	1719	572	N
AtIQD31	AT1G74690.1	28061159-28064625	1	1764	587	?
AtIQD32	AT1G19870.1	6895015-6898725	1	2385	794	N
AtIQD33	AT5G35670.1	13856103-13858059	5	1329	442	?
Rice						
OsIQD1	LOC_Os05g03190.1	81361–85365	5	1425	474	?
OsIQD2	LOC_Os01g61720.8	106290–110032	1	1503	500	N
OsIQD3	LOC_Os03g44610.1	118442–126461	3	1323	440	N
OsIQD4	LOC_Os03g57330.1	48853–52578	3	1269	422	?
OsIQD5	LOC_Os01g09790.1	152586–155207	1	1329	442	?
OsIQD6	LOC_Os05g38790.1	23568–26042	5	1617	538	N
OsIQD7	LOC_Os06g06160.1	90441–94332	6	1062	353	N
OsIQD8	LOC_Os12g42430.3	133867–139274	12	1329	442	?
OsIQD9	LOC_Os01g51230.1	7612–9986	1	1326	441	?
OsIQD10	LOC_Os05g46350.1	37570–40338	5	1227	408	?
OsIQD11	LOC_Os05g46350.1	15479–17371	1	1692	563	?
OsIQD12	LOC_Os06g23380.1	18921–22322	6	1413	470	N
OsIQD13	LOC_Os02g19640.1	59894–65564	2	1458	485	?
OsIQD14	LOC_Os08g02250.1	88345–90355	8	1632	543	?
OsIQD15	LOC_Os04g48160.1	151253–153796	4	1395	464	N
OsIQD16	LOC_Os03g43580.1	144176–145684	3	1344	447	?
OsIQD17	LOC_Os03g06570.1	135566–136967	3	1254	417	?
OsIQD18	LOC_Os10g28420.1	17834–19903	10	1458	485	?
OsIQD19	LOC_Os05g44630.1	24971333–24973284	5	1104	367	N
OsIQD20	LOC_Os01g53980.1	95943–99625	1	1737	578	?

OsIQD21	LOC_Os05g09520.1	45436–47015	5	1494	497	?
OsIQD22	LOC_Os01g09470.1	70239–72382	1	1398	465	N
OsIQD23	LOC_Os05g45930.1	54441–58756	5	1725	574	N
OsIQD24	LOC_Os01g51840.1	38561–44222	1	1725	574	N
OsIQD25	LOC_Os04g56740	85710–91604	4	2682	893	N
OsIQD26	LOC_Os03g04210	57690–58691	3	912	303	N
OsIQD27	LOC_Os04m04570	27592940–27594955	4	1107	368	?
OsIQD28	LOC_Os06g40850.1	329–2234	6	1209	432	N
Brachypodium						
BdIQD1	Bradi4g01360.2	869983-875532	4	1308	435	N
BdIQD2	Bradi2g05680.1	4160734-4162695	2	1482	493	M
BdIQD3	Bradi2g05840.1	4350695-4353358	2	1320	439	M
BdIQD4	Bradi1g06350.1	4255837-4260120	1	1248	415	N
BdIQD5	Bradi1g13650.3	10494438-10501428	1	1260	419	N
BdIQD6	Bradi3g13710.1	12184580-12187933	3	1752	583	C
BdIQD7	Bradi1g14090.1	10991279-10993725	1	1392	463	C
BdIQD8	Bradi2g18220.1	16134114-16136622	2	1308	435	N
BdIQD9	Bradi2g18640.2	16526685-16532110	2	1731	576	C
BdIQD10	Bradi5g18767.1	21961423-21963974	5	1356	451	N
BdIQD11	Bradi2g19410.1	17078761-17081761	2	1107	368	N
BdIQD12	Bradi3g26990.1	27763162-27766280	3	1431	476	N
BdIQD13	Bradi2g33370.1	33463747-33465745	2	1419	472	N
BdIQD14	Bradi1g36140.1	31926928-31929055	1	1311	436	N
BdIQD15	Bradi2g37980.2	38342608-38346663	2	1425	474	N
BdIQD16	Bradi2g47867.1	48270948-48274013	2	1281	426	N
BdIQD17	Bradi2g48210.2	48526630-48531648	2	1665	554	N
BdIQD18	Bradi1g49070.2	48055425-48059038	1	1023	340	N
BdIQD19	Bradi2g49490.1	49598263-49600689	2	1173	390	N
BdIQD20	Bradi2g54170.2	53142697-53147456	2	1479	492	N
BdIQD21	Bradi2g57567.1	55747039-55750930	2	1734	577	C
BdIQD22	Bradi1g62220.1	61752750-61756461	1	1467	491	N
BdIQD23	Bradi1g74410.1	71905417-71907676	1	1233	410	N
Populus						
PtIQD1	Potri.001G021000	1607742–1610595	1	1395	464	C
PtIQD2	Potri.001G108800	8650631–8655082	1	1434	477	N
PtIQD3	Potri.001G190500	17147397–17151292	1	1236	411	N
PtIQD4	Potri.001G215200	21798241–21805654	1	1314	437	M
PtIQD5	Potri.001G375700	39064696–39069035	1	909	302	N
PtIQD6	Potri.001G448100	48222177–48224324	1	1389	462	N
PtIQD7	Potri.002G025200	1586081–1591447	2	2445	814	N
PtIQD8	Potri.002G178500	13811073–13813833	2	1671	556	N
PtIQD9	Potri.003G042700	5706743–5709706	3	1332	443	N
PtIQD10	Potri.003G047800	6990533–6993068	3	1230	409	N
PtIQD11	Potri.003G122800	14483539–14488038	3	1431	476	C
PtIQD12	Potri.004G053000	4153576–4157021	4	939	312	C
PtIQD13	Potri.005G051500	3687165–3689335	5	1014	337	N
PtIQD14	Potri.005G106300	8150665–8151156	5	408	135	N
PtIQD15	Potri.005G236200	24358919–24365526	5	2460	819	N
PtIQD16	Potri.006G046000	3264556–3268258	6	1296	431	N
PtIQD17	Potri.006G131100	10742805–10746955	6	1440	479	N
PtIQD18	Potri.006G131100	23817184–23820667	6	1257	418	N
PtIQD19	Potri.008G156700	10615675–10618176	8	1308	435	N
PtIQD20	Potri.009G015800	2662464–2669160	9	1332	443	N
PtIQD21	Potri.010G082600	10880956–10883713	10	1293	430	N
PtIQD22	Potri.010G218100	20440978–20445678	10	1470	489	N
PtIQD23	Potri.011G063200	5770289–5773023	11	939	312	N
PtIQD24	Potri.011G096500	11757480–11761074	11	912	303	M
PtIQD25	Potri.011G153400	17201694–17204132	11	1659	552	N
PtIQD26	Potri.012G016200	1564728–1567623	12	1398	465	C
PtIQD27	Potri.012G022500	1980877–1985207	12	1716	571	N
PtIQD28	Potri.012G069900	9347701–9354581	12	1863	620	C
PtIQD29	Potri.012G139800	15401076–15406028	12	1368	455	C
PtIQD30	Potri.013G038900	2660734–2662179	13	591	196	N

PtIQD31	Potri.013G127200	13984689–13990617	13	1587	528	N
PtIQD32	Potri.014G104600	8221204–8223802	14	1677	558	C
PtIQD33	Potri.015G005900	367350–369625	15	1554	517	N
PtIQD34	Potri.015G012500	804149–807576	15	1383	460	E.R
PtIQD35	Potri.015G063600	8766252–8772461	15	1779	592	C
PtIQD36	Potri.015G142200	14886292–14891121	15	1356	451	C
PtIQD37	Potri.016G086300	6805977–6809919	16	1437	478	N
PtIQD38	Potri.017G079600	9337863–9340813	17	1422	473	N
PtIQD39	Potri.018G062000	7657258–7659644	18	1242	413	N
PtIQD40	Potri.019G095700	12681872–12687989	19	1593	530	N
tomato						
SUN		SL2.40ch07-2395262	7	1226	421	N
SlSUN1	Solyc10g079240.1.1	60140568-60142797(+)	10	1226	421	N
SlSUN2	Solyc01g009340	3537789-3541754(+)	1	1521	506	N
SlSUN3	Solyc01g088250.2.1	74824669-74827406(+)	1	1377	458	N
SlSUN4	Solyc01g097490	80081666-80082520(+)	1	1233	410	N
SlSUN5	Solyc02g077260.2.1	36828296-36831764(-)	2	909	302	N
SlSUN6	SL1.00sc00090_96	41309977-41310589(+)	2	387	128	M
SlSUN7	Solyc02g087760.2.1	44643248-44648496(+)	2	1671	556	N
SlSUN8	Solyc03g026110.2.1	7899519-7903299(-)	3	1461	486	N
SlSUN9	Solyc03g026440.2.1	9052195-9054923(+)	3	561	186	N
SlSUN10	Solyc03g026110.2.1	46466257-46469172(-)	3	1410	469	N
SlSUN11	Solyc03g121760.2.1	63883128-63885625(-)	3	1290	429	N
SlSUN12	Solyc04g016480.2.1	7305326-7308804(-)	4	1233	410	N
SlSUN13	Solyc04g016480.2.1	44467392-44470126(-)	4	1185	394	N
SlSUN14	Solyc04g081210.2.1	62803374-62809165(+)	4	2589	862	N
SlSUN15	Solyc05g007130.2.1	1694652-1699497(+)	5	1656	551	N
SlSUN16	Solyc06g052010.1.1	32163038-32167768(+)	6	1194	397	N
SlSUN17	Solyc06g053450.2.1	32744659-3275125(+)	6	1779	592	N
SlSUN18	Solyc06g066430.2.1	38090484-38092311(-)	6	1179	392	N
SlSUN19	Solyc08g007920.1.1	2425707-2427454(+)	8	705	234	N
SlSUN20	Solyc08g007930.1.1	2436580-2438211(+)	8	684	227	N
SlSUN21	Solyc08g014280.2.1	3966331-3970317(+)	8	1620	539	N
SlSUN22	Solyc08g062940.2.1	49582645-49584764(+)	8	930	309	N
SlSUN23	Solyc08g080470.2.1	60928845-60932810(+)	8	1500	499	N
SlSUN24	Solyc08g083240.2.1	62923263-62925544(-)	8	1470	489	N
SlSUN25	Solyc09g007410.2.1	985216-988218 (-)	9	1452	483	N
SlSUN26	Solyc09g082560.2.1	63677616-63679791(-)	9	1404	467	N
SlSUN27	Solyc10g005000.2.1	4134-5923 (-)	10	1185	394	N
SlSUN28	Solyc10g008790.2.1	2859728-2865652(-)	10	915	304	N
SlSUN29	Solyc10g084280.1.1	63223672-63226246(+)	10	1437	478	N
SlSUN30	Solyc10g086060.1.1	64348811-64350952(-)	10	1416	471	N
SlSUN31	Solyc11g071840.1.1	64348811-64350952(+)	10	1347	448	N
SlSUN32	Solyc12g008520.1.1	1931971-1934807(+)	12	1230	409	N
SlSUN33	Solyc12g014130.1.1	4940650-4946027(+)	12	786	261	N
soybean						
GmIQD1	Glyma01g01030	681417–683646	1	1263	420	N
GmIQD2	Glyma01g05100	4750065–4755456	1	1692	563	N
GmIQD3	Glyma01g42620	53843322–53846963	1	1191	396	N
GmIQD4	Glyma02g00710	502944–506146	1	1254	417	N
GmIQD5	Glyma02g02370	1778568–1785636	2	1692	563	N
GmIQD6	Glyma02g15590	14083370–14089609	2	1608	535	N
GmIQD7	Glyma03g33560	41092523–41096935	2	1434	477	N
GmIQD8	Glyma03g40630	46330165–46332185	3	1125	374	N
GmIQD9	Glyma04g02830	2030287–2036251	3	2715	904	N
GmIQD10	Glyma04g05520	4187757–4190317	4	1353	450	N
GmIQD11	Glyma04g23760	27192306–27195532	4	1353	450	N
GmIQD12	Glyma04g34150	40144241–40151603	4	1752	583	N
GmIQD13	Glyma04g41380	47220698–47225472	4	1392	463	N
GmIQD14	Glyma05g01240	785189–792757	4	1761	586	N
GmIQD15	Glyma05g03450	2638386–2641896	5	1338	445	N
GmIQD16	Glyma05g35920	39871246–39873985	5	1128	375	N
GmIQD17	Glyma06g02841	1950849–1956820	5	2532	843	N

GmIQD18	Glyma06g05530	10606168–10611219	6	1353	450	E.R
GmIQD19	Glyma06g13470	3957759–3960421	6	1341	446	N
GmIQD20	Glyma06g20341	16752231–16759304	6	1755	584	N
GmIQD21	Glyma07g01040	607467–610485	6	1302	433	N
GmIQD22	Glyma07g01760	1164144–1167157	7	1191	396	N
GmIQD23	Glyma07g05680	4335391–4339373	7	1641	546	N
GmIQD24	Glyma07g14910	14801071–14803234	7	1398	465	C
GmIQD25	Glyma07g32531	37416802–37421879	7	873	290	N
GmIQD26	Glyma07g32860	37753882–37759623	7	1602	533	N
GmIQD27	Glyma08g03710	2630927–2633769	7	1311	436	N
GmIQD28	Glyma08g20430	15453660–15456579	8	1266	421	N
GmIQD29	Glyma08g21430	16271106–16273575	8	1209	402	N
GmIQD30	Glyma08g40880	40742659–40748073	8	1644	547	C
GmIQD31	Glyma09g26630	33163730–3316945	8	1449	482	C
GmIQD32	Glyma09g30780	37552192–37557238	9	1305	434	N
GmIQD33	Glyma09g35920	41794962–41798738	9	1407	468	N
GmIQD34	Glyma10g00630	386683–389158	10	1272	423	N
GmIQD35	Glyma10g05720	4477640–4481520	10	1425	474	N
GmIQD36	Glyma10g35721	43974896–43978361	10	1452	483	N
GmIQD37	Glyma10g38310	46118444–46123432	10	1395	464	C
GmIQD38	Glyma10g39030	46764292–46767407	10	1410	469	N
GmIQD39	Glyma11g20880	17714458–17717939	10	1374	457	N
GmIQD40	Glyma12g01410	842971–846738	11	1383	460	N
GmIQD41	Glyma12g31610	35181013–35188577	12	1269	422	N
GmIQD42	Glyma12g35711	38833825–38837834	12	885	294	N
GmIQD43	Glyma13g20070	23539750–23543840	12	1413	470	N
GmIQD44	Glyma13g24070	27399608–27404534	13	774	257	N
GmIQD45	Glyma13g30590	33154582–33158861	13	900	299	N
GmIQD46	Glyma13g34700	36237460–36241896	13	1173	390	N
GmIQD47	Glyma13g38800	39521853–39528595	13	1278	425	N
GmIQD48	Glyma13g42440	42441870–42445047	13	1239	412	N
GmIQD49	Glyma13g43031	42796469–42804226	14	1143	380	N
GmIQD50	Glyma14g11050	9335703–9339095	14	1254	417	N
GmIQD51	Glyma14g25860	31470493–31475301	15	1377	458	N
GmIQD52	Glyma15g02370	1595640–1598698	15	1137	378	N
GmIQD53	Glyma15g02940	2051157–2053854	15	1251	416	C
GmIQD54	Glyma15g08660	6125483–6129362	16	927	308	N
GmIQD55	Glyma16g02240	1759053–1762330	16	1653	550	N
GmIQD56	Glyma16g22935	26564269–26565120	16	426	141	C
GmIQD57	Glyma16g32161	35337880–35343544	17	1434	477	C
GmIQD58	Glyma17g10660	8002515–8009332	17	1767	588	N
GmIQD59	Glyma17g14000	10763173–10767584	17	1344	447	N
GmIQD60	Glyma17g23770	23932487–23938307	17	1386	461	N
GmIQD61	Glyma17g34520	38500561–38503843	18	1242	413	N
GmIQD62	Glyma18g16130	16440695–16446996	19	1644	547	N
GmIQD63	Glyma19g36270	43610551–43615073	19	1434	477	N
GmIQD64	Glyma19g43300	48995941–48998264	20	1113	370	N
GmIQD65	Glyma20g28800	37708013–37709907	20	1434	477	N
GmIQD66	Glyma20g29550	38392614–38397440	20	1371	456	C
GmIQD67	Glyma20g31810	40423269–40426995	20	1470	489	C

Table S2. The major MEME motif sequences and lengths in *PeIQDs* proteins.

Motif	Width	Coserved amino acid sequences
1	41	EWAAVKIQTAFRGYLARRALRALKGIVKLQALVRGHLVRKQ
2	28	PSFPNYMANTESAKAKTRCQSAPRQRPD
3	29	NHFFYDPNDPNWGWNWLERWMIICPWENR
4	21	ATTLHCMQALVRFQAQVRARR
5	41	MGEGWCDSIGSVEEIQAMLISRQEAAIKRERAMAYAFSHQW
6	15	MGKAMKWIKSVLFGK
7	41	EKHKRNPRCFTSVPEDNVPDSQLSELEKVKRNLNKVTNSMA
8	41	HRRRLSDGGDSNYERSPRIVEMDTCHLRCRSTRITSRYYAP
9	41	VTDGKPHTRKASYAMETESGKLKRNVRKCSAMTVEPFQTNM
10	50	NWVTTGEKVNEPTVFAPAVEIMPLQDIDNEDNVLGNDEEHRSKEEP LSNG

Table S3. Summary of the duplicated gene-pairs and determination of Ka and Ks values of the IQD family in moso bamboo , rice and *Brachypodium*.

	Ka	Ks	Ka/Ks	Duplication date (MY)
paralogous genes				
PeIQD2-PeIQD29	0.0095	0.108	0.88	8.31
PeIQD4-PeIQD9	0.0978	0.1267	0.77	9.75
PeIQD5-PeIQD26	0.0486	0.1259	0.39	9.68
PeIQD10-PeIQD12	0.0723	0.1467	0.49	11.28
PeIQD11-PeIQD16	0.0156	0.1106	0.14	8.51
PeIQD14-PeIQD24	0.0285	0.1078	0.26	8.29
PeIQD18-PeIQD21	0.0573	0.0696	0.82	5.35
PeIQD19-PeIQD23	0.0745	0.1522	0.49	11.71
PeIQD20-PeIQD25	0.0582	0.151	0.39	11.62
orthologous genes				
BdIQD7-PeIQD29	0.4951	0.5457	0.91	41.99
BdIQD22-PeIQD3	0.1295	0.3274	0.4	25.81
OsIQD2-PeIQD28	0.0686	0.4269	0.16	32.84
OsIQD28-PeIQD22	0.1808	0.4878	0.37	37.52
OsIQD17-PeIQD7	0.3037	0.4114	0.74	31.6
OsIQD26-PeIQD27	0.1181	0.2601	0.45	20
OsIQD22-PeIQD13	0.106	0.2842	0.37	21.86

Table S4. **The full length of PeIQD8 and PeCaM2 cDNAs.**

	length of cDNAs
PeIQD12	ATGGGGCGTGCCATGAGATGGCTCAAGAAGCTACTGACAGGCAGGAAGGGC<br>GGGCACAAAGGCCTCAAGGAGAACCAGGTCGCAAGCGACTGGCGTAACGGG<br>ACGGAGAGGGAGGAGAGGTGGATCTTTGTGAAGCAGCGGAAGAGCGGAGTT<br>GATGGCGGCAGGCGGCCCTCGGAGGCCGCGGCGGCGGCGGCGGCGGA<br>GCCGTCGCAGGTCAGGCCATGCCATTGTGGTGAGGAAGATGCCAGACCTCG<br>CGAGGACGAGGCGGCCGTCGTGATCCAGAAAGCCTTCAGGGGCTACCTGGC<br>TAGGAAAGCGCTTCGCGCTCTCGTAGCACTTGTCAAGCTACAGGCTCTAGTC<br>CGAGGCTACCTTGTGAGGAAGAAAGTCGCCACGACTCTACACAGGTTGCAG<br>GCGCTCATGCGGCTGCAGGCCGATTCTTACGTCAAGAGAGCTTCCTACCGGA<br>AATCCATGGAACAAGAGAGGATCATCACGCAAGAAGCCCGGGTGAAGCCGT<br>CGGCGACGCCGGCGCACCGTCGGAGGCTCTCCGACAGCACGGACTCCAACT<br>ACGAGCGCAGCCCGCGAATCGTCGAGATGGACACGTGCCACCTCCGCTCCC<br>GGTCCACCCGGATCGTGAGCAGCCGGTACGCCCCCGACCACTCGTCGGACCG<br>CCTTACCCCGGTCCTCGCGCCGTCGTGCTCGCCGGTGTCCAGCAAGCAGCCC<br>CCACGGCTCTCAATCCAGCGGTGCCGCGAGCGGGACCCCAGGCAAGCCAAG<br>ACTGCGCAGAACACGCCTCGCTGCTTCGTGCCCGCTCCGCCGCACGACTTGC<br>CCGCCAAGAGCGTCGACGGCCTGACGTCGCGGCGGCTCTCGCACCGGGACG<br>TGATCGTCAGCCCGCGCTACATGGAGGATACGGCCTCGTCCGCGGCGAGGA<br>CGCAGTGCCAGAGCGCGCCGAGGCAGCGGCAGGCCGAGGCGCCGCCGAGG<br>GCGAGCCTGACCCGGTCCGGGTCGAGGAAGTCGTGCTCGCAGATGCATGAC<br>AGCGCCTTCTGCTTCCAGTGCTCGGTTGCCACCCAGACGGGCTGCTCCGAGC<br>TCAGCGACGAGGCGGCCAGAGATTACTACCTGGACAGGATGTGGTGA
PeCaM2	ATGGCGGACCAGCTCACCGACGAGCAGATCGCCGAGTTCAAGGAGGCCTTC<br>AGCCTCTTCGACAAGGACGGCGACGGTTGCATCACTACTAAGGAGCTTGGA<br>ACCGTGATGCGATCCCTTGGGCAGAACCCTACTGAGGCAGAGCTGCAGGAC<br>ATGATCAATGAGGTTGATGCTGATGGCAATGGGACCATCGACTTCCCAGAGT<br>TCCTGAACCTGATGGCAAGGAAGATGAAGGATACCGACTCTGAGGAGGAGC<br>TCAAGGAGGCCTTCCGTGTCTTTGACAAGGACCAGAACGGTTTCATCTCGGC<br>TGCTGAGCTCCGCCATGTCATGACCAACCTTGGGGAGAAGCTGACTGATGAG<br>GAAGTTGATGAGATGATCCGCGAGGCCGATGTGGATGGCGATGGCCAGATC<br>AACTATGATGAGTTCGTCAAGGTCATGATGGCCAAGTGA

Table S5. Summary of abiotic-stress inducible cis-elements is in the promoter regions of IQD genes in moso bamboo. Cis-elements with larger numbers are highlighted in bold. Motif S000153 (CCGAC) overlaps with motif S000402 (ACCGAC) and S000418 (RCCGAC), and motif S000415 ACGT overlaps with motif S000414 (ACGTG) and S000133 (CCACGTGG), so the total number of motifs was based on the specific position of motifs on the promoter regions.

**Abiotic stress**	**Gene name** / ***cis*-element**	**motif sequence**	***PeIQD1***	***PeIQD3***	***PeIQD4***	***PeIQD5***	***PeIQD6***	***PeIQD7***	***PeIQD27***
Drought-stress	S000133	CCACGTGG	0	0	0	0	0	0	0
	S000153	CCGAC	0	**5**	1	2	1	1	**9**
	S000174	CACATG	0	2	1	2	0	1	1
	S000175	CTAACCA	0	0	0	0	0	2	1
	S000176	CNGTTR	4	2	1	3	**8**	0	2
	S000177	TAACTG	2	1	0	0	1	0	0
	S000402	ACCGAC	0	0	0	0	0	0	0
	S000408	WAACCA	3	3	2	2	3	3	3
	S000413	CATGTG	0	2	1	2	0	0	1
	S000414	ACGTG	1	2	0	2	4	2	**7**
	S000415	ACGT	**6**	**10**	4	**6**	**8**	**10**	**18**
	S000418	RCCGAC	0	0	1	0	0	1	4
	total		16	27	12	17	25	20	46
Wound-stress	S000244	AACGTGT	0	0	0	0	0	0	0
	S000444	AGATCCAA	0	0	0	0	0	0	1
	S000457	TGACY	2	1	2	3	4	**13**	**6**
	total		2	1	2	3	4	13	7

Abiotic stress	Gene name / *cis*-element	motif sequence	***PeIQD 8***	***PeIQD 9***	***PeIQD 10***	***PeIQD 11***	***PeIQD 28***	***PeIQD 12***	***PeIQD 13***
Drought -stress	S000133	CCACGTGG	0	0	0	0	0	0	0
	S000153	CCGAC	2	2	0	0	1	**5**	0
	S000174	CACATG	0	1	1	0	1	2	3
	S000175	CTAACCA	0	0	0	0	0	0	0
	S000176	CNGTTR	**5**	4	**6**	4	**7**	**9**	4
	S000177	TAACTG	1	0	0	0	0	1	0
	S000402	ACCGAC	2	1	0	0	1	0	0
	S000408	WAACCA	1	4	**5**	1	3	1	2
	S000413	CATGTG	0	1	1	0	1	2	3
	S000414	ACGTG	3	2	3	1	2	2	**5**
	S000415	ACGT	**6**	**8**	**18**	1	**14**	**6**	**22**
	S000418	RCCGAC	2	1	0	4	1	4	0
	total		22	24	34	11	31	32	39
Wound-stress	S000244	AACGTGT	0	0	0	0	0	0	0
	S000444	AGATCCAA	0	0	0	0	0	1	0
	S000457	TGACY	**7**	**15**	**5**	2	**6**	**5**	**9**
	total		7	15	5	2	6	6	9

**Abiotic stress**	**Gene name / *cis*-element**	**motif sequence**	***PeIQD 14***	***PeIQD 15***	***PeIQD 16***	***PeIQD 17***	***PeIQD 18***	***PeIQD 19***	***PeIQD 29***
Drought-stress	S000133	CCACGTGG	0	0	0	0	0	0	0
	S000153	CCGAC	1	3	4	3	**6**	**5**	1
	S000174	CACATG	2	0	2	0	0	3	3
	S000175	CTAACCA	1	2	0	0	2	0	1
	S000176	CNGTTR	4	3	3	3	**8**	**5**	**7**
	S000177	TAACTG	2	0	0	1	0	0	0
	S000402	ACCGAC	0	0	1	1	4	0	1
	S000408	WAACCA	1	3	1	2	**6**	0	3
	S000413	CATGTG	1	0	2	0	0	3	3
	S000414	ACGTG	2	0	1	0	4	1	**5**
	S000415	ACGT	**10**	4	4	4	0	**8**	**16**
	S000418	RCCGAC	1	3	2	1	4	2	1
	total		25	18	20	15	34	27	41
Wound-stress	S000244	AACGTGT	0	0	0	0	1	1	0
	S000444	AGATCCAA	0	0	0	0	0	0	0
	S000457	TGACY	2	0	**8**	3	**6**	**8**	**7**
	total		2	0	8	3	7	9	7

**Abiotic stress**	**Gene name** / ***cis*-element**	**motif sequence**	***PeIQD 21***	***PeIQD 20***	***PeIQD 22***	***PeIQD 23***	***PeIQD 24***	***PeIQD 25***
Drought-stress	S000133	CCACGTGG	0	0	0	0	0	0
	S000153	CCGAC	3	1	4	3	4	2
	S000174	CACATG	0	3	3	3	2	1
	S000175	CTAACCA	0	0	0	0	1	0
	S000176	CNGTTR	4	**5**	**6**	**9**	**5**	3
	S000177	TAACTG	0	0	2	1	2	1
	S000402	ACCGAC	0	0	3	0	3	0
	S000408	WAACCA	1	3	1	3	1	4
	S000413	CATGTG	0	3	3	3	2	1
	S000414	ACGTG	4	**6**	**7**	2	**5**	3
	S000415	ACGT	**16**	**14**	**16**	4	**14**	**6**
	S000418	RCCGAC	0	0	4	0	3	2
	total		28	35	49	28	42	23
Wound-stress	S000244	AACGTGT	0	0	0	0	0	0
	S000444	AGATCCAA	0	0	0	0	0	0
	S000457	TGACY	2	**5**	**7**	**5**	**6**	**9**
	total		2	5	7	5	6	9

Table S6. Primers used in the paper..

Primers for qRT-PCR analysis

Gene	Orientation	Primer Sequence(5'->3')	Length	Tm	GC%	Product length	Efficiency values
PeIQD1	Forward	GGCCGCGCACGATAGA	16	59.2	42.8	144	92%
	Reverse	TCTTGATGGGAGAAGGCATAGG	22	60.1	50		
PeIQD2	Forward	GCGGGTAGGTGGCTCAGAA	19	61.9	40.91	17	105.60%
	Reverse	GATTGGCTGTCGGCTTTGTC	20	59.8	55.6		
PeIQD3	Forward	AACCGAAGCAGAAACCGAAAC	21	58	47.6	158	110.10%
	Reverse	CTTCCCTGCCAGCCATCTC	19	61.9	47.8		
PeIQD4	Forward	GAGGCATGAGGGTGCAATTAA	21	58	47.6	97	91.10%
	Reverse	GCTGCTGGTGAGAAAGAGCAT	21	60	52.4		
PeIQD5	Forward	GGCAGGCTGCAGAATCATTAC	21	60	52.4	124	90.30%
	Reverse	GATGTGCCCGGACTAGACCTT	21	61.9	54.5		
PeIQD6	Forward	TGACTGGGTTTGGGTTCTTTG	21	55.2	33.33	118	103.60%
	Reverse	GAACAACGCAAGCATTTGCTAA	22	61.64	52.38		
PeIQD7	Forward	ACCTAGTGGGCATTTTGGATTC	22	58.2	45.5	119	101.70%
	Reverse	CTTCCCAAATTCGTTTCCGTATC	23	58.4	43.5		
PeIQD8	Forward	CGGGACTCCAACCCTGATAA	20	59.8	55	169	105.50%
	Reverse	GGCAGGACGGGTCAGTTTT	19	59.7	51.9		
PeIQD9	Forward	TGGGAGAGTCGCCTTATGGA	20	59.8	55	95	92.50%
	Reverse	CTGAACACCGCGAATTGGT	19	57.6	52.6		
PeIQD10	Forward	CGGGACGGAGAGGGAGAA	18	61.9	41.67	136	93%
	Reverse	GCTGTCAACTCCGCTCTTCCT	21	61.9	39.13		
PeIQD11	Forward	AAGAGCAAGCAGCCGTCAAG	20	59.8	55	116	107.50%
	Reverse	CCCGACGTGCCAGATAACC	19	61.9	53.2		
PeIQD12	Forward	CGCCACGACTCTACACAGGTT	21	61.9	41.7	135	109.70%
	Reverse	GTAGGAAGCTCTCTTGACGTAAGAATC	27	62	44.4		
PeIQD13	Forward	CAGCGTTTGCTTTGATTGGA	20	60.32	45.83	165	104.20%
	Reverse	CCCTTGGCTCAATCTTGCA	19	59.06	50		
PeIQD14	Forward	CCGTCAGTCCCAAGCTACATG	21	61.9	51.5	134	94.10%
	Reverse	CCCTGGAGGCGGGACTTA	18	61.9	46.7		
PeIQD15	Forward	GTGGATGGAGGCATGATGTG	20	59.8	41.67	110	108.30%
	Reverse	GGTTCTGGCAGTGAAACTGGTAA	23	60.2	47.8		
PeIQD16	Forward	AAGAGCAAGCAGCCGTCAA	19	57.6	52.6	157	108.10%

	Reverse	TGCCCGACGAGCCAGATA	18	59.6	51.8		
PeIQD17	Forward	AACAATGAGCCTTTTGAAATCCA	23	54.8	34.8	126	103.90%
	Reverse	GGAGCTATGCGATCAGTTTTGG	22	60.1	50		
PeIQD18	Forward	TTCGTTCCCCACCAAATCA	19	55.4	47.45	144	110.00%
	Reverse	CCTTGGCGGAACTGGAGTATT	21	60	50		
PeIQD19	Forward	GCCCAGGCTCGTGTCAGA	18	61.9	45	129	106.80%
	Reverse	TTTTCCGTGCCACTTGGTTT	20	55.8	50		
PeIQD20	Forward	TTTTCCGTGCCACTTGGTTT	18	55	57.9	140	94.20%
	Reverse	GCAGCGACAACGGCTTTAC	19	59.7	50		
PeIQD21	Forward	CTGCCTTGCCATTCATTGTG	20	57.8	45	142	90.50%
	Reverse	TCCATGCATCCGACGATTTA	20	55.8	48.6		
PeIQD22	Forward	CGCGGTCACAGGTCTTCAG	19	61.9	52	127	91.30%
	Reverse	CTCATCCTCGACACGTTGGA	20	59.8	55.6		
PeIQD23	Forward	CTGCTGTCAAGCGCGAAA	18	57.3	57.9	99	91.50%
	Reverse	GAACCAGGTTGCCGCTGAT	19	59.7	45.54		
PeIQD24	Forward	CCAGCCCCCCGTCAA	15	59	40	241	107.10%
	Reverse	TTCACAAGGATTGGTGTCACTTTAG	25	58.7	37.5		
PeIQD25	Forward	CGGCCGTCATGATTCAGAA	19	57.6	52	169	103.40%
	Reverse	ACAAGTGATTTGAGAGCACGAAGT	24	58.6	41.7		
PeIQD26	Forward	TCGAACCATAAGGATCTTGTGAAAT	25	57.1	36	80	108.90%
	Reverse	TGCAGCCTGCCTCCTAACTAG	21	61.9	57.1		
PeIQD27	Forward	CCCAAACGAACTGGAAATGTG	21	58.1	45	139	106.50%
	Reverse	ACCCCGGACCCAATAACAG	19	61.9	55		
PeIQD28	Forward	CCCGTGCCATCTCAATCC	18	59.6	51.4	118	91.90%
	Reverse	CTCGGTGGACGGCTTGAC	18	61.9	56.7		
PeIQD29	Forward	GGACCGTCATCGGCATTG	18	59.6	61.1	118	107.20%
	Reverse	GAGCCTTCCTCGCCAGGTA	19	61.9	63.2		
		**Primers for Yeast two-hybrid assay**					
PeIQD8	Forward	GGGGTACCATGTTGAGTTACAGGCCTGAATG					
	Reverse	GC**TCTAGA**TCATTTGTCGCTGCTTCCAC					
		**Primers for site-specific mutagenesis**					
PeIQD8Dell112-113	Forward	GTAACCTCGGAAGGCGGTCTTTATTGCTGCAAG					
	Reverse	CTGGCAAGAAGAGCACTGCGAGCATTAAGAGG					
PeIQD8I112T	Forward	CTGGCAAGAAGAGCACTGCGAGCATTAAGAGG					
	Reverse	GTAACCTCGGAAGGCGGTTTGGGTCTTTATTGC					
